# Hyperactivity is a Core Endophenotype of Elevated Neuregulin-1 Signaling in Embryonic Glutamatergic Networks

**DOI:** 10.1093/schbul/sbab027

**Published:** 2021-04-19

**Authors:** Tilmann Götze, Maria Clara Soto-Bernardini, Mingyue Zhang, Hendrik Mießner, Lisa Linhoff, Magdalena M Brzózka, Viktorija Velanac, Christian Dullin, Fernanda Ramos-Gomes, Maja Peng, Hümeyra Husseini, Eva Schifferdecker, Robert Fledrich, Michael W Sereda, Katrin Willig, Frauke Alves, Moritz J Rossner, Klaus-Armin Nave, Weiqi Zhang, Markus H Schwab

**Affiliations:** 1 Department of Neurogenetics, Max-Planck-Institute of Experimental Medicine, Goettingen, Germany; 2 Cellular Neurophysiology, Hannover Medical School, Hannover, Germany; 3 Laboratory of Molecular Psychiatry, Department of Mental Health, Westfälische Wilhelm-University of Münster, Münster, Germany; 4 Department of Neurology, University Medicine Göttingen (UMG), Göttingen, Germany; 5 Department of Psychiatry, Ludwig-Maximilian-University Munich, Munich, Germany; 6 Institute for Diagnostic and Interventional Radiology, University Medical Center, Goettingen, Germany; 7 Translational Molecular Imaging, Max-Planck-Institute of Experimental Medicine, Goettingen, Germany; 8 Italian Synchrotron “Elettra,"Trieste, Italy; 9 Institute of Anatomy, University of Leipzig, Leipzig, Germany; 10 Center for Nanoscale Microscopy and Molecular Physiology of the Brain, University Medical Center Göttingen, Göttingen, Germany; 11 Max Planck Institute of Experimental Medicine, Göttingen, Germany; 12 Department of Neuropathology, University Hospital Leipzig, Leipzig, Germany

**Keywords:** schizophrenia, conditional transgenic mice, ErbB4 receptor, ventricular enlargement

## Abstract

The neuregulin 1 (NRG1) ErbB4 module is at the core of an “at risk” signaling pathway in schizophrenia. Several human studies suggest hyperstimulation of NRG1-ErbB4 signaling as a plausible pathomechanism; however, little is known about the significance of stage-, brain area-, or neural cell type-specific NRG1-ErbB4 hyperactivity for disease-relevant brain endophenotypes. To address these spatiotemporal aspects, we generated transgenic mice for Cre recombinase-mediated overexpression of cystein-rich domain (CRD) NRG1, the most prominent NRG1 isoform in the brain. A comparison of “brain-wide” vs cell type-specific CRD-NRG1 overexpressing mice revealed that pathogenic CRD-NRG1 signals for ventricular enlargement and neuroinflammation originate outside glutamatergic neurons and suggests a subcortical function of CRD-NRG1 in the control of body weight. Embryonic onset of CRD-NRG1 in glutamatergic cortical networks resulted in reduced inhibitory neurotransmission and locomotor hyperactivity. Our findings identify ventricular enlargement and locomotor hyperactivity, 2 main endophenotypes of schizophrenia, as specific consequences of spatiotemporally distinct expression profiles of hyperactivated CRD-NRG1 signaling.

## Introduction

Schizophrenia is a neuropsychiatric disorder with a significant but complex genetic component.^1^ According to the neurodevelopmental hypothesis of schizophrenia, genetically imparted perturbations of brain development contribute to disease manifestation in young adults.^2,3^ However, stage-, area-, or neural cell type-specific pathomechanisms that link genetic risk factors with disease-relevant brain endophenotypes are not well defined.

Variants of the human *NRG1* and *ERBB4* genes are plausible genetic risk factors for schizophrenia.^4–6^*NRG1* encodes a family of alternatively spliced isoforms that serve as epidermal growth factor (EGF) like ligands for the receptor tyrosine kinase ErbB4. Human NRG1 isoforms belong to 6 different groups (I–VI), based on distinct N-terminal protein domains. NRG1 isoforms from group III contain an N-terminal cysteine-rich domain (CRD) that serves as a second transmembrane domain. As a consequence, CRD-NRG1 isoforms mediate juxtacrine signaling, even after proteolytic processing.^7^

NRG1/ErbB4 signaling regulates various neurodevelopmental processes, in particular, the migration of cortical interneurons, synapse formation, and myelination,^8^ consistent with a NRG1/ErbB4 signalopathy as a plausible component of schizophrenia etiopathophysiology.^9^ Some insight into the nature of this signalopathy came from human studies using post mortem brain tissue, which found evidence for increased NRG1 and ErbB4 expression, as well as ErbB4 receptor hyperactivation in schizophrenia patients.^10–12^ Notably, individuals inheriting a schizophrenia risk haplotype located in the regulatory region of the *NRG1* gene (HapICE) exhibit an increased mRNA expression for CRD-NRG1^13^, suggesting a pathogenic role for hyperactive juxtacrine CRD-NRG1/ErbB4 signaling in schizophrenia.

To test this concept, we and others have investigated transgenic mouse lines with constitutive overexpression of the CRD-NRG1 isoform under control of the Thy1.2 or CamKII promoter and found several brain endophenotypes with relevance for schizophrenia, including ventricular enlargement, impaired prepulse inhibition (PPI), anxiety-like behavior, and deficits in working memory.^14–16^ Together, these studies imply hyperactive CRD-NRG1/ErbB4 signaling as an attractive candidate for a major signalopathy in schizophrenia. However, considering complex and dynamic expression patterns in the brain,^17^ constitutive overexpression in available CRD-NRG1 transgenic mice has impeded the precise mapping of brain endophenotypes to spatiotemporally distinct CRD-NRG1 signaling dysfunctions. Thus, in support of informed future therapeutic strategies, we generated a mouse model for the conditional activation of juxtacrine CRD-NRG1 signaling in the brain.

## Materials and Methods

### Transgenic Mice

The *Stop-Nrg1* transgene was generated by cloning murine HA-NRG1 type IIIβ1a cDNA^18^ via *Spe*I/*Xho*I restriction sites into a cassette containing the chicken β-actin promoter, EGFP flanked by 2 loxP sites, and a bovine growth hormone polyA site in a pBluescriptKS vector. Transgene was excised via *Sal*I and injected into C57BL/6N oocytes. One founder was backcrossed to C57BL/6N. All experiments were performed in compliance with animal policies of the MPI-EM and approved by the Federal State of Lower Saxony (license numbers #33.9-42502-04-10/0288; #33.19-42502-04-16/2340). Genotyping of genomic DNA from tissue biopsies was performed according to manufacturer´s instructions (1-Step Tissue and Cells genomic DNA isolation kit, nexttec^T^) with PCR primers: F5’-GGTGGCTATAAAGAGG-TCATCAG-3’; R5’-GTCCACAAATACCCACTTTAGGCCAGC-3’. Genotyping of *NEX-Cre* and *CamKII-Cre* mice was as described.^19,20^

### Immunohistochemistry

Organ fluorescence was examined with a Leica MZ16F fluorescence stereomicroscope (488 nm excitation). Images were processed with FIJI software.^21^ Fluorescent immunostaining of paraplast embedded brain was described.^18^ Chromogenic immunostaining was conducted as above, except for peroxidase inactivation for 5 min with 3% H_2_0_2_ prior to blocking. Cryotome and vibratome sections were immunostained following permeabilization and blocking with 4% horse serum, 0.1 % Triton X-100 in 1× PBS for 30 min at RT. For antibodies and visualization of immunoreactivity, see supplementary material. Images were acquired using a Zeiss AxioZ brightfield microscope (Carl Zeiss Jena, Germany). Z-stacks were acquired using a Leica TCS SP2 microscope (Leica Microsystems GmbH, Wetzlar, Germany). Images were processed using Zeiss Zen, ImageJ,^22^ and Adobe Photoshop. For the quantification of immunostainings, see^23^ and supplementary material.

### µCT Scans

Mice were sacrificed by cervical dislocation and decapitated. Dissected brains were rinsed 5× in water and transferred to 35% and 70% ethanol for 1 h each. Brains were fixed and stained at RT in 4% PFA in PBS, pH 7.4 and 0.7% PTA in 70% ethanol for 8 days under constant agitation and stored in 70% ethanol. For µCT analysis, brains were embedded in 4% agarose. Samples were scanned using QuantumFX in vivo µCT (Perkin Elmer), nanotome µCT (Phoenix GE), or eXplore Locus SP µCT (Trivoil), resulting in 3D data sets with isotropic voxel sizes ranging from 40 µm to 5 µm. Brain and ventricle sizes were quantified in 3D using a region growing algorithm implemented in SCRY v4.0 software (Kuchel & Sautter GbR). The region growing function was used for volume tracing in virtual Z-stacks in 3D. Data were analyzed (GraphPad Prism software) by 2-tailed *t*-tests or one-way ANOVA. For immunostaining and quantifications of post µCT brains see supplementary material.

### Biochemistry

Protein extract preparation was performed as described.^14^ Proteins were separated on 8–15% SDS-polyacrylamide gels, blotted onto polyvinylidene difluoride (PVDF) membranes (Hybond-P, Invitrogen) and visualized according to manufacturer’s instructions (Western Lightning Plus-ECL, Perkin Elmer Life Science, Inc.). For details and antibodies, see supplementary material. Membranes were scanned using an Intas ChemoCam Imager. Densitometric analysis was conducted using FIJI software. Statistical significance was tested by one-way ANOVA with Bonferroni’s multiple comparison test and *t*-test (GraphPad Prism 5.0).

### Electrophysiology

Recordings were performed on hippocampal slices in standard recording solutions. Data acquisition and analysis were done as described^14,15^ (see supplementary methods for details).

### Behavior

Behavioral testing and statistical analysis were conducted as described^14,15,24,25^ (see supplementary methods for detail).

## Results

### A Transgenic Mouse Model for Conditional Hyperstimulation of CRD-NRG1/ErbB4 Signaling

**Fig. 1.**
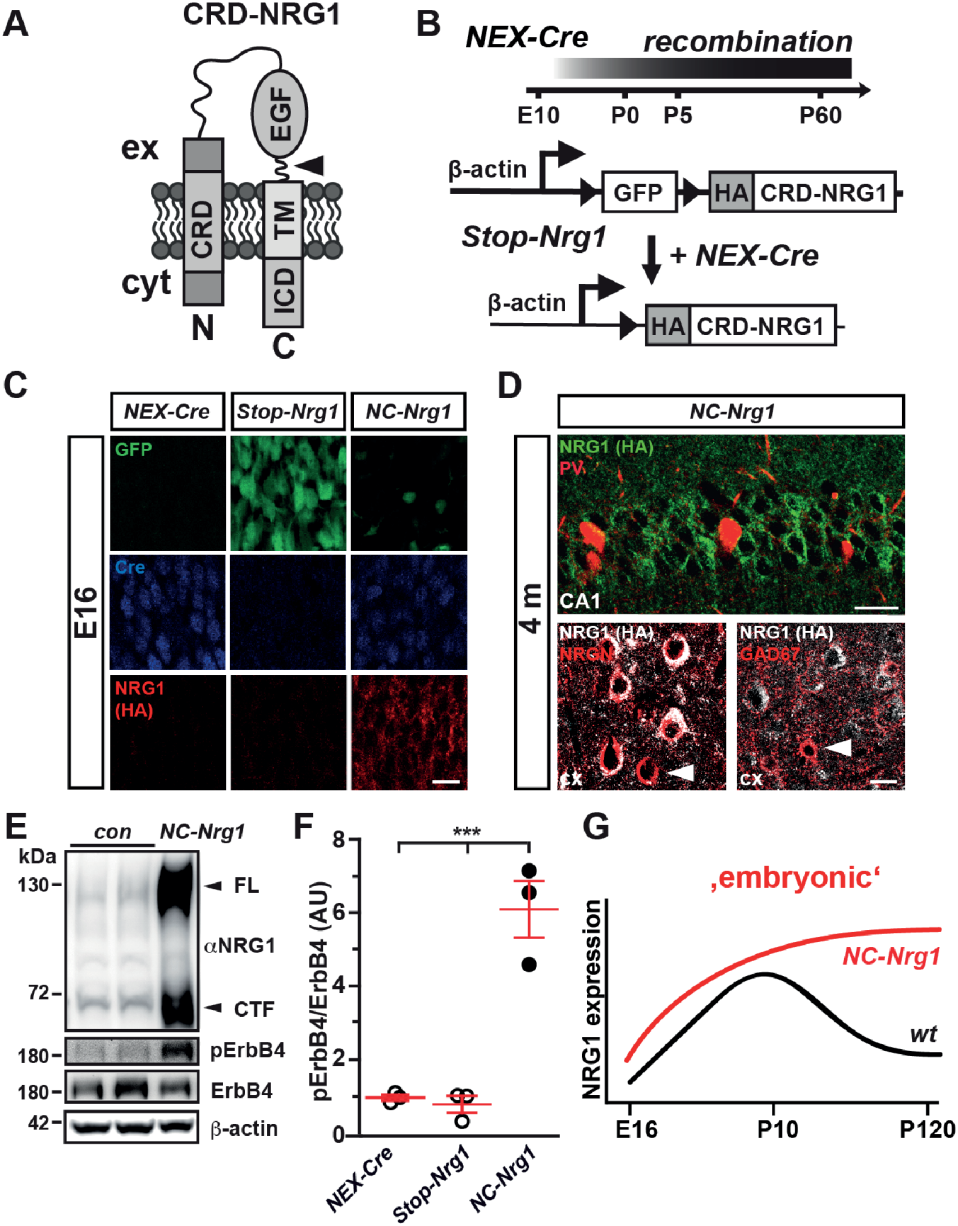
Glutamatergic network-specific stimulation of CRD-NRG1/ErbB4 signaling. (A) CRD-NRG1 protein structure. Arrowhead, proteolytic cleavage in juxtamembrane “stalk” region. C, C-terminus; CRD, cysteine rich domain; cyt, cytoplasm; EGF, epidermal growth factor-like domain; ex, extracellular; ICD, intracellular domain; N, N-terminus; TM, transmembrane domain. (B) (top) Schedule of Cre-mediated recombination in *NEX-Cre* mice. (bottom) *NEX-Cre*-mediated removal of “floxed” *Stop* element permits HA-CRD-NRG1 expression from *Stop-Nrg1* transgene. (C) HA-CRD-NRG1 expression in the cortical plate of *NC-Nrg1* mice. Fluorescent immunostainings on coronal brain sections (E16). Scale bar, 10 µm. (D) HA-CRD-NRG1 expression in hippocampus and cortex is restricted to glutamatergic projection neurons. Immunostaining on brain sections from *NC-Nrg1* mice (4 months) for HA epitope, glutamatergic (neurogranin, NRGN), and GABAergic markers (parvalbumin, PV; GAD67). Arrowhead (left panel) marks NeuN^+^ neuron without HA epitope expression (most likely GABAergic neuron). Arrowhead (right panel) points to HA epitope-negative GABAergic neuron. cx, cortex. Scale bars, 20 µm. (E) Western blotting of hippocampal protein lysates from controls (*NEX-Cre*; *Stop-Nrg1*) and *NC-Nrg1* mice (4 months). CTF, C-terminal fragment; FL, full length. (F) Densitometric quantification of pErbB4 bands (Tyr1284). Integrated density values normalized to ErbB4/β-actin. *n* = 3 each, ***P < .001; one-way ANOVA with Bonferroni’s multiple comparison test (F (2) = 41.62, P = .0003). (G) Schematic temporal profile of HA-CRD-NRG1 overexpression in *NC-Nrg1* mice.

CRD-NRG1 harbors 2 transmembrane domains (figure 1A) and signals to ErbB4, its main receptor in the brain. Here, we generated conditional transgenic mice (*Stop-Nrg1*) for Cre-mediated expression of N-terminally hemagglutinin (HA) epitope-tagged CRD-NRG1^26^ (figure 1B; supplementary figures 1A and 1B). Based on “Stop” element-encoded GFP, we observed β-actin promoter-driven transgene expression in the brain and various organs (supplementary figure 1C; data not shown). Immunostaining in *Stop-Nrg1* mice (in the absence of a Cre “driver”) demonstrated transgene (GFP) expression in glutamatergic neurons, oligodendrocytes, and spinal cord α−motoneurons. However, only a small subset of GABAergic interneurons and astrocytes expressed the *Stop-Nrg1* transgene (supplementary figure 1D; data not shown).

Cortical CRD-NRG1 expression peaks during embryonic and postnatal stages.^17^ To model CRD-NRG1 overexpression in the embryonic cortex, we crossbred *Stop-Nrg1* mice to *NEXCre* driver mice,^19^ in which Cre-mediated recombination is initiated at embryonic day (E) 12 in postmitotic glutamatergic neurons (figure 1B). Immunostaining identified moderate transgenic CRD-NRG1 expression in the cortical plate of NEXCre*Stop-Nrg1 double transgenic mice (referred to as *NC-Nrg1*) at E16 (figure 1C). We also produced a model for CRD-NRG1 overexpression in glutamatergic neurons starting at postnatal stages by breeding to *CamKII-Cre* driver mice^20^ (supplementary figure 2A). Resulting double transgenic mice (*CC-Nrg1*) and *NC-Nrg1* mice displayed glutamatergic neuron-specific HA-CRD-NRG1 expression in the brain (figure 1D; supplementary figures 2B and 2C). Western blotting demonstrated CRD-NRG1 overexpression in the hippocampus and neocortex of *NC-Nrg1* and *CC-Nrg1* mice, which was associated with hyperphosphorylated ErbB4 receptor (figures 1E and 1F; supplementary figures 2D and 2E). In conclusion, the expression profile of CRD-NRG1 in *NC-Nrg1* mice (figure 1G) allows to address the impact of a developmentally hyperstimulated schizophrenia risk pathway on adult brain functions.

**Fig. 2.**
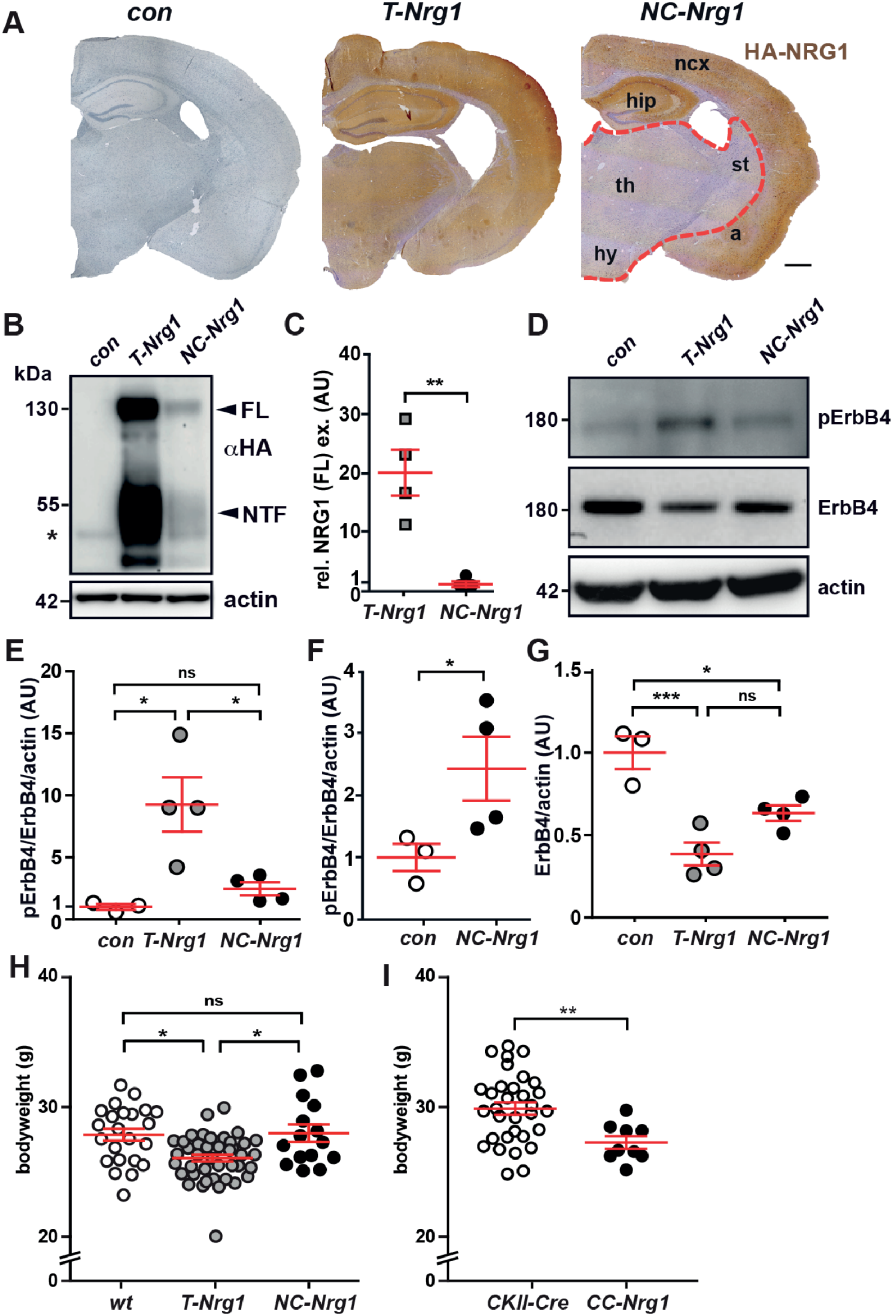
Cortical-restricted vs brain-wide CRD-NRG1-mediated ErbB4 hyperactivation in transgenic mice. (A) Immunostainings for HA-CRD-NRG1 (age 4 months). Scale bar, 500 μm. a, amygdala; hip, hippocampus; hy, hypothalamus; ncx, neocortex; st, striatum; th, thalamus. (B) Western blotting of cortical protein lysates (P10). Actin served as loading control. Asterisk, unspecific band. FL, full-length; NTF, N-terminal fragment. (C) Densitometric quantification of full-length CRD-NRG1 normalized to actin. *n* = 3; **P < .01. *t*-test. AU, arbitrary units. (D) Western blot analysis of pErbB4 (Tyr1284) and ErbB4 in cortical protein lysates (P10). Actin served as loading control. (E–G) Densitometric quantification of (E, F) pErbB4 bands normalized to ErbB4 and actin (F (2) = 9.515, P = .0077), (G) ErbB4 bands normalized to actin (F (2) = 18.18, P = .0011). *n* = 3–4; *P < .05, ***P < .001. One-way ANOVA with Bonferroni’s multiple comparison test, *t*-test; AU, arbitrary units. (H) Body weight (*wt, T-Nrg1, NC-Nrg1;* 3 months) (F (2) = 7.937, P = .0007). (I) Body weight of *CC-Nrg1* mice (15–18 weeks). Values, mean ± SEM. **P < .01. One-way ANOVA with Bonferroni’s multiple comparison test.

We previously generated “constitutive” transgenic mice, which overexpress HA-CRD-NRG1 under control of the Thy1.2 promoter^18^ (*T-Nrg1*). Immunostaining confirmed cortical and subcortical CRD-NRG1 expression in *T-Nrg1* transgenic mice, whereas overexpression in *NC-Nrg1* mice was limited to cortical projection neurons (figure 2A). Western blotting at P10 revealed higher cortical CRD-NRG1 expression in *T-Nrg1* compared to *NC-Nrg1* mice (figures 2B and 2C). In line, cortical ErbB4 phosphorylation was increased in both lines; however, it was ~4-fold higher in *T-Nrg1* compared to *NC-Nrg1* mice (figures 2D–2F). Furthermore, we observed a corresponding reduction of total ErbB4 protein in *NC-Nrg1* and *T-Nrg1* mice (figures 2D and 2G). These findings indicate that a comparative analysis of these mouse lines could be employed to inform about expression level-, brain region-, and neural cell type-specific CRD-NRG1 functions.

### Uncoupling of CRD-NRG1 Overexpression From Body Weight Reduction, Ventricular Enlargement, and Neuroinflammation in *NC-Nrg1* Mice

Intraperitoneal injection of mice with the recombinant EGF-like domain of NRG1 (NRG1β) slows weight gain.^27^ We observed reduced body weight in adult *T-Nrg1* mice (figure 2H), and in additional lines with Thy1.2 promoter-driven “brain-wide” expression of full-length CRD-NRG1 (line *SMDA*^28^; supplementary figure 2G) and a variant that mimics BACE1 processed “active” CRD-NRG1 (*T-Nrg1∆*^18^; supplementary figures 2H, 2I, and 2K). In case of *T-Nrg1∆* mice, reduced body weight was already detectable at P20 (supplementary figure 2K), consistent with increased CRD-NRG1∆ signaling potency. Body weight was unaltered in *NC-Nrg1* mice (figure 2H; supplementary figure 2J) but reduced in *CC-Nrg1* mice (figure 2I), which harbor substantial subcortical CRD-NRG1 expression, including the hypothalamus (supplementary figure 2B). Together, these findings support a subcortically located CRD-NRG1 function in body weight control.

**Fig. 3.**
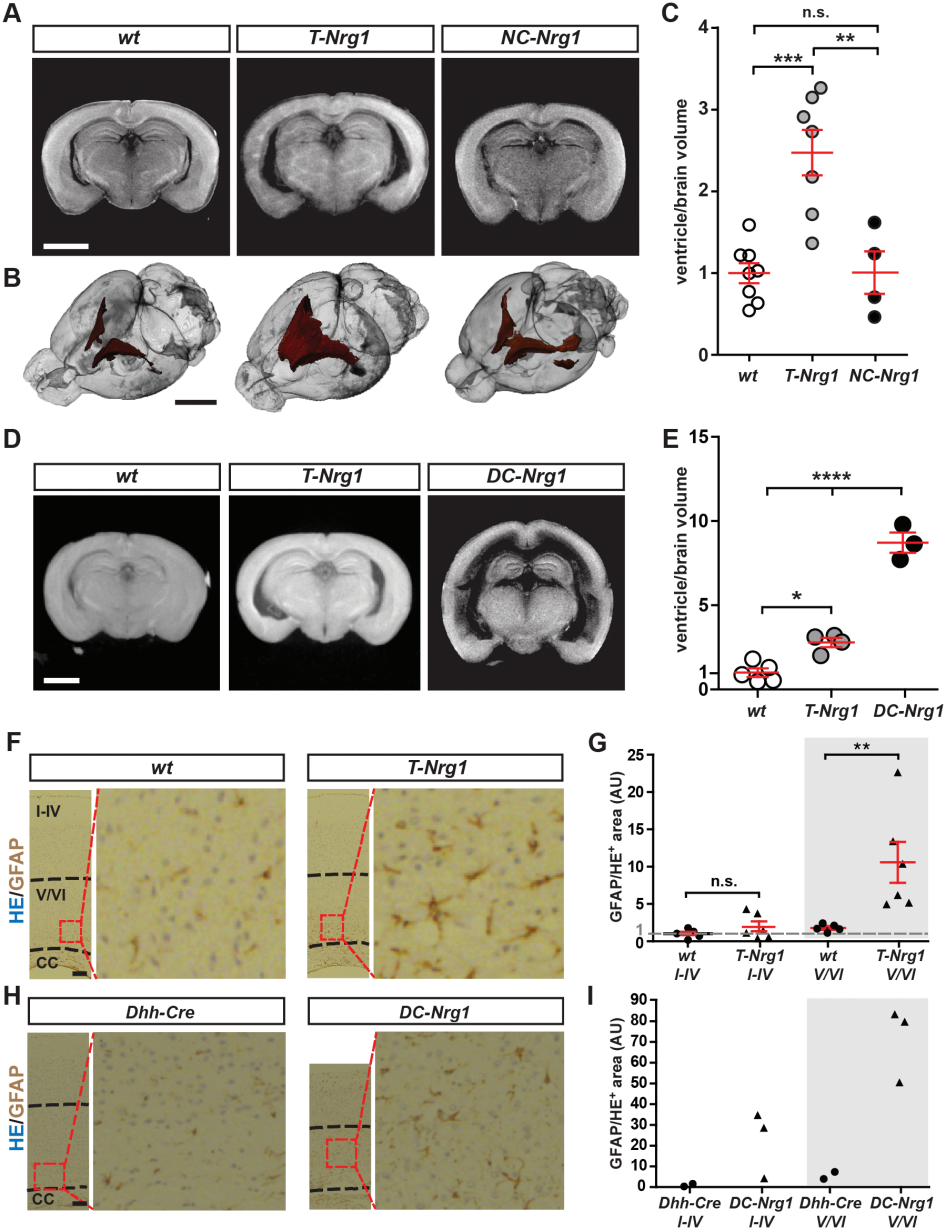
CRD-NRG1 signals that affect the ventricular system originate from cells other than glutamatergic neurons. (A) µCT analysis (3 months). (B) Volume rendering of µCT data sets. (C) Quantification of total ventricular volume/brain volume normalized to wt (F (2) = 14.13, P = .0004). Error bars, normalized mean ± SEM. One-way ANOVA with Bonferroni’s multiple comparison test. *P < .05; ***P < .001. Scale bars, 1 mm. (D) µCT analysis of *T-Nrg1* mice (P35) and *DC-Nrg1* mice (P30). (E) Quantification of ventricular volume as in (C) (F (2) = 119.9, P < .0001); ****P < .0001. (F) H&E histology and GFAP immunostaining on coronal brain sections following µCT analysis. (left) Upper dashed line separates cortical layers I–IV from V/VI, lower line marks border to corpus callosum (cc). (right) Zoomed images of boxed area in left panel. Scale bar, 100 µm. (G) GFAP^+^ area (normalized to HE^+^ area) calculated as ratio lower/upper cortex. Values normalized to wt. Error bars, normalized mean ± SEM. Mann–Whitney *U*-test, **P < .01. (H) GFAP immunostaining of brain sections following µCT analysis. Upper dashed line in *DC-Nrg1* sample separates equally sized cortical areas. Scale bar, 100 µm. (I) Quantification as in (H). Statistical analysis not performed due to *n* = 2 *Dhh-Cre* controls.

Mice from the *SMDA* line display ventricular enlargement.^14^ Ex vivo microcomputed tomography (µCT) confirmed a similar ventricular enlargement in *T-Nrg1* mice at 3 months (figures 3A–3C) and P37 (figures 3D and 3E). Ventricular enlargement was most pronounced for lateral ventricles but also involved the foramen of Monroe (supplementary figures 3A–3D). Ventricular size was unaltered in *NC-Nrg1* mice (figure 3A–3C), but breeding *Stop-Nrg1* mice to a *Desert hedgehog (Dhh)-Cre* driver line^29^ with recombination in a subset of cortical neurons, astrocytes, and endothelial cells (supplementary figure 3E) resulted in massively increased ventricles in Dhh-Cre*Stop-Nrg1 mice (*DC-Nrg1*) at 1 month (figures 3D and 3E; supplementary figure 3F). Immunostaining showed no signs of axonal swellings, T-cell infiltration, activated microglia or astrogliosis in *NC-Nrg1* mice (supplementary figures 3G–3I; data not shown). In contrast, the somatosensory cortex of *T-Nrg1* and *DC-Nrg1* mice harbored increased GFAP signals in cortical layers V/VI (figures 3F–3I), consistent with neocortical astrogliosis.

These findings identify ventricular enlargement as a specific outcome of hyperactive CRD-NRG1 signaling and suggest that causative pathogenic signals originate outside glutamatergic neurons. Together, *NC-Nrg1* mice provide a new mouse model to investigate the impact of moderate, glutamatergic network-specific CRD-NRG1/ErbB4 hyperstimulation on brain functions in the absence of major confounding endophenotypes.

### *NC-Nrg1* Mice Display Reduced Inhibitory Neurotransmission, Impaired LTP, and Abnormal Theta Oscillations

**Fig. 4.**
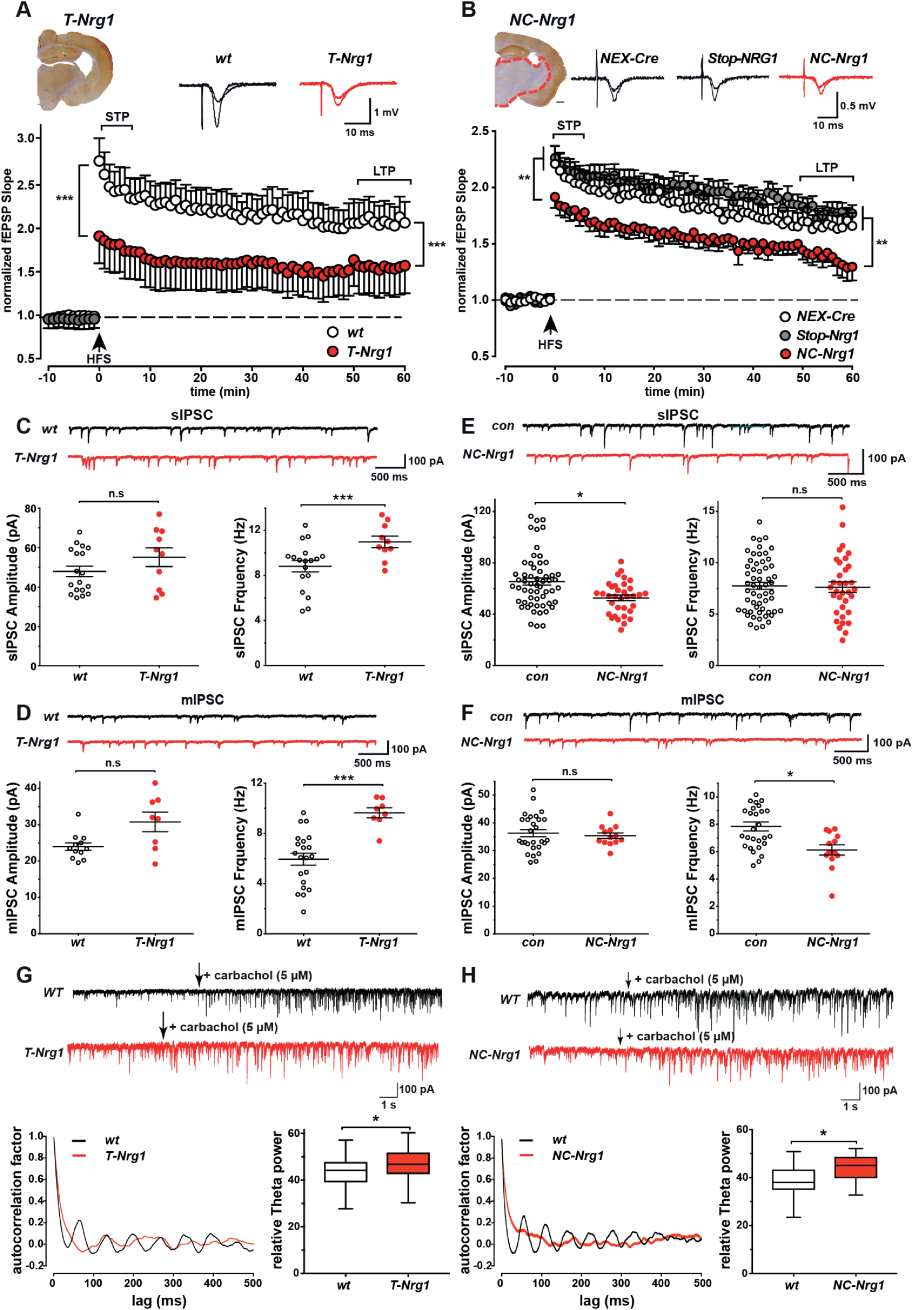
Inhibitory synaptic transmission, synaptic plasticity, and theta oscillations are affected in *T-Nrg1* and *NC-Nrg1* mice. (A, B) Top: sample traces of responses before and after HFS in CA1 of *T-Nrg1* (A) and *NC-Nrg1* mice (B). Below: reduced LTP in *T-Nrg1* (A) and *NC-Nrg1* mice (B). (C) Top: sIPSC traces in PFC of *WT* and *T-Nrg1* mice; below: averaged sIPSC frequency was increased in PrL of *T-Nrg1* mice. (D) Top: mIPSC traces in the PFC of *WT* and *T-Nrg1* mice; below: increased averaged mIPSC frequency in PrL of *T-Nrg1* mice. (E) Top: sIPSC traces in PFC of control and *NC-Nrg1* mice; below: reduced averaged sIPSC amplitude in PrL of *NC-Nrg1*. (F) Top: mIPSC traces in PFC of control and *NC-Nrg1 mice*; below: averaged mIPSC frequency was reduced in PrL of *NC-Nrg1* mice. Bars, group means (±SEM). *p < .05; **p < .01; ***p < .001. (G, H) Top: recordings of sIPSCs in a CA1 pyramidal cell before and following addition of 5 µM carbachol in *T-Nrg1* (G) and *NC-Nrg1* mice (H). Below: autocorrelation and relative power of 10-s stretches from recordings after carbachol treatment in *T-Nrg1* (G) and *NC-Nrg1* mice (H).

Consistent with our findings in SMDA mice,^14^ LTP at the Schaffer collateral (SC) CA1 synapse was reduced and inhibitory synaptic transmission in prefrontal cortex (PFC) was increased in *T-Nrg1* mice (figures 4A, 4C, and 4D). Hippocampal LTP was also reduced in *NC-Nrg1* mice (figure 4B), whereas fEPSP slopes and paired-pulse ratios in CA1, and glutamatergic neurotransmission in PFC were unaltered in both lines (supplementary figure 4). In contrast to *T-Nrg1* mice, inhibitory neurotransmission was reduced in the PFC of *NC-Nrg1* mice (figures 4E and 4F). Furthermore, carbachol-induced theta oscillations were impaired and the relative theta power was increased in CA1 of both lines (figures 4G and 4H). These data show that a physiologically relevant chronic increase in glutamatergic CRD-NRG1 expression in *NC-Nrg1* mice reduces, not increases, inhibitory neurotransmission in the PFC, and impairs synaptic plasticity and theta oscillations in CA1.

These synaptic and network dysfunctions could result from alterations in glutamatergic and/or GABAergic neurons. To identify structural changes in glutamatergic networks, we examined dendritic spines of CA1 pyramidal cells in stratum radiatum by STED microscopy but found no evidence for profoundly altered spine density or shape in *T-Nrg1* mice at 10 weeks of age (supplementary figures 5A–5C). Consistent with our studies in *SMDA* and *T-Nrg1* mice,^14^ PV^+^ interneuron number was unaltered in the hippocampus (supplementary figures 4D and 4E) but moderately reduced in layer 5 of the somatosensory cortex of NC-Nrg1 mice (supplementary figure 4D–4G). Thus, changes observed in GABAergic neurotransmission in the PFC of NC-Nrg1 mice could result from a reduced density of PV^+^ synapses.

### CRD-NRG1 Overexpression in Glutamatergic Cortical Networks Induces Hyperactivity

*T-Nrg1* mice display increased anxiety, impaired PPI, and working memory deficits.^14,15^ To obtain a corresponding behavioral profile for *NC-Nrg1* mice, we performed a behavioral test battery. A basic physical exam (including testing of eyelid, whisker, and ear reflexes and grip strength) revealed no obvious deficits in parental lines and *NC-Nrg1* mice (supplementary table 1). Also pain sensitivity in the hot plate test was not affected in *NC-Nrg1* mice (supplementary figure 6A) in line with intact sensory functions (that critically depend on NRG1 signaling^30,31^).

In contrast to *T-Nrg1* mice, *NC-Nrg1* mice showed no signs of anxiety in the light-dark preference test (supplementary figure 6B) and open field test, with time spent in the center of the arena and number of rearings similar to controls (supplementary figures 6C and 6D). Furthermore, while *NC-Nrg1* mice displayed a subtle reduction in the startle response prior conditioning (supplementary figure 6F), PPI, and working memory, as assessed in the Y-maze, were not altered (supplementary figures 6G and 6H).

**Fig. 5.**
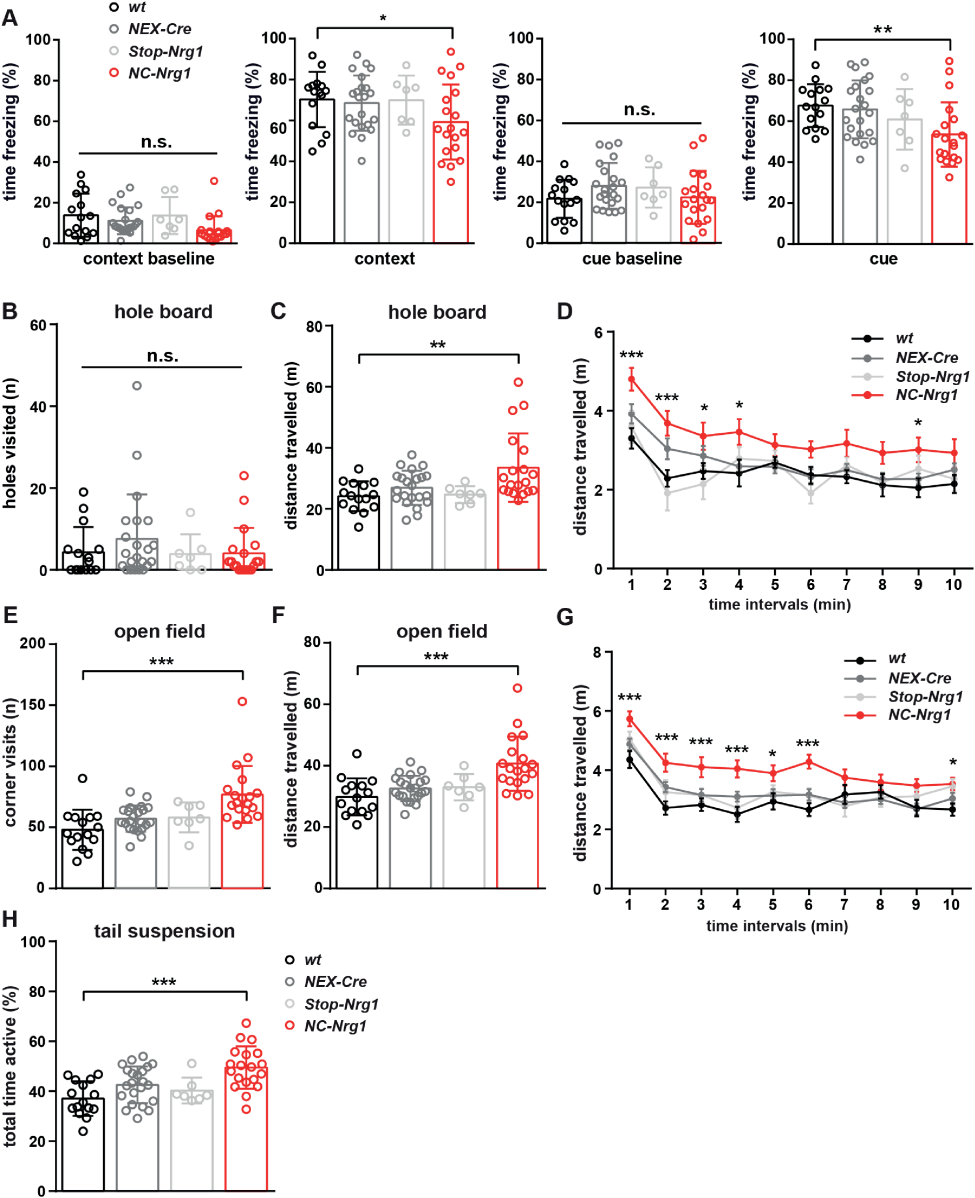
Cortical-restricted CRD-NRG1 hyperstimulation in glutamatergic neurons causes novelty-induced hyperactivity. (A) Tendency for reduced freezing of *NC-Nrg1* mice when tested for cued fear memory in fear conditioning (context baseline, F (3) = 2.035, P = .1188; context, F (3) = 2.798, P = .0479; cue baseline, F (3) = 1.390; P = .2546; cue, F (3) = 3.674, P = .017). (B–D) Hole board test. Number of visits was unaltered (F (3) = 0.8934, P = .45). (B–D) Hole board test. Number of visits was unaltered (F (3) = 0.8934, P = .45) (B), but distance traveled was increased (F (3) = 5.385, P = .0024) (C), most pronounced during early stages when distance was plotted against time (F (3) = 26.99, P < .0001) (D). (E–G) Open field test. Number of corners visited (F (3) = 9.073, P < .0001) (E) and distance traveled (F (3) = 9.519; P < .0001) (F) were increased, with increased distance traveled most pronounced during early stages (F (3) = 39.09, P < .0001) (G). (H) *NC-Nrg1* mice exhibited increased total time active in the tail suspension test (F (3) = 8.343, P = .0001). Data were analyzed using ordinary one-way ANOVA with Sidak’s multiple comparisons posttest (B, C; E, F, H), 2-way ANOVA with Tukey’s posttest (A) or 2-way ANOVA with Dunnett’s posttest for repeated measurements (D, G). Individual data points are shown with means ±SD (A–C, E, F, H) or ±SEM (D, G) (for color figure refer online version). n.s., not significant; ***P < .001, **P < .01, *P < .05.

Of note was moderately reduced freezing of *NC-Nrg1* mice when exposed to context and auditory cue in fear conditioning (figure 5A). However, reduced freezing behavior in *NC-Nrg1* mice may be attributed to increased motor activity. Whereas in the hole board test, the number of holes visited and exploration time were unaltered (figure 5B; data not shown), *NC-Nrg1* mice showed an increased distance traveled compared to controls (figure 5C). This was particularly visible in the first minutes of the hole board test (figure 5D). We also observed hyperactivity in the open field test, where *NC-Nrg1* mice showed an increased time active (supplementary figure 6E), number of corner visits (figure 5E), and distance traveled (figure 5F). Again, *NC-Nrg1* mice were more active during early stages, consistent with novelty-induced locomotor hyperactivity (figure 5G). Finally, *NC-Nrg1* mice exhibited increased time active in the tail suspension test compared to controls (figure 5H), most likely reflecting general hyperactivity, instead of increased motivation to escape from the aversive situation. These findings suggest locomotor hyperactivity as a core endophenotype of moderately increased CRD-NRG1 signaling in cortical glutamatergic networks.

## Discussion

To investigate the genetic component of the neurodevelopmental hypothesis of schizophrenia, we modeled moderate CRD-NRG1 overexpression with an embryonic onset in cortical glutamatergic neurons in *NC-Nrg1* mice. Compared with constitutive *T-Nrg1* mice, *NC-Nrg1* mice provide a physiologically more relevant hyperstimulation of CRD-NRG1/ErbB4 signaling in the neocortex and hippocampus, supporting their employment to examine region- and cell type-specific CRD-NRG1 functions in the brain.

Intraperitoneal NRG1β injection reduces weight gain in mice,^27^ raising the question of peripheral vs central NRG1 functions in body weight control.^32,33^ Body weight was unaltered in *NC-Nrg1* mice but reduced in *T-Nrg1* mice and conditional *CC-Nrg1* mice. This identifies a central, most likely subcortical, CRD-NRG1 function in the control of body weight, possibly via autocrine signaling to ErbB4^+^ neurons in the hypothalamus.^33,34^ This hypothesis could be tested by breeding *Stop-Nrg1* mice to a Cre-driver line for hypothalamic recombination.^35^

Ventricular enlargement is a major neuroanatomical biomarker in schizophrenia.^36^ It has been linked to NRG1 risk variants in first-episode schizophrenia patients^37^ and in individuals at high risk for psychosis.^38^ We reported enlarged lateral ventricles in transgenic mice with CRD-NRG1 overexpression.^14^ Here, we confirm increased ventricular size in *T-Nrg1* mice, demonstrating ventricular enlargement as a specific outcome of hyperactive CRD-NRG1 signaling. Ventricular size was massively increased in *DC-Nrg1* mice but unaltered in *NC-Nrg1* mice. This indicates that pathogenic CRD-NRG1 signals affecting the ventricular system derive from cells other than glutamatergic neurons, eg, ependymal cells,^39^ possibly causing abnormal autocrine NRG1/ErbB4 signaling, ciliopathy, and altered cerebrospinal fluid circulation.^40^

Hyperactivity is a core symptom in schizophrenia,^41,42^ but its neurobiological underpinnings are only partially understood. We found locomotor hyperactivity in *NC-Nrg1* mice across several behavioral test paradigms. In particular, *NC-Nrg1* mice displayed novelty induced hyperactivity, a rodent correlate of positive symptoms of schizophrenia^43^ in the open field and hole board test. Unaltered working memory and PPI in conditional *NC-Nrg1* mice (with moderate ErbB4 hyperstimulation) suggest that previously reported deficits in these domains in *T-Nrg1* mice^14,15^ result from more pronounced and/or spatially distinct (in particular subcortical) CRD-NRG1 dysfunctions in constitutive *T-Nrg1* mice.

Hyperactivity in *NC-Nrg1* mice is consistent with reduced motor activity in conditional NRG1 loss-of-function mutants using the *CKII-Cre* driver line.^14^ However, hyperactivity was also reported in heterozygous “global” NRG1 and ErbB4 loss-of-function mutants^44–46^ but absent in constitutive transgenic mice with CRD-NRG1 overexpression under control of the CamKII promoter.^16^ These discrepancies between our conditional and previous constitutive genetic approaches most likely emerge from different timing and/or the spatial extend of genetic manipulations in different models and underscore the importance of a precise isoform-, region-, and cell type-specific mapping of NRG1 dysfunctions in the brain.

How does hyperactivity emerge in *NC-Nrg1* mice? We suggest synergistic reciprocal interactions between glutamatergic neuron-derived CRD-NRG1 overexpression and changes in GABAergic and dopaminergic neurotransmission via PV^+^/ErbB4^+^ interneurons. This notion is supported by our finding of reduced inhibitory neurotransmission in *NC-Nrg1* mice and hyperactive motor behavior in several genetic and pharmacological models of GABAergic impairment, including mouse mutants with disrupted NMDA receptor function in interneurons.^47–50^ Ultimately, GABAergic deficits could elicit secondary glutamatergic hyperexcitability, cortical network desynchronization, and hyperactivity in *NC-Nrg1* mice. Preclinical animal studies also provide a strong link between locomotor hyperactivity and abnormal dopaminergic signaling,^51^ and infusion of recombinant NRG1β into rat brains was shown to stimulate dopamine release in the hippocampus, presumably indirectly via D4 receptors on PV^+^ interneurons.^52^ Thus, chronically increased CRD-NRG1 signaling may promote altered dopaminergic signaling in the hippocampus and neocortex, which in concert with GABAergic changes may induce locomotor hyperactivity, as well as abnormal hippocampal LTP^52^ and oscillatory activity.^53^

Together, our data identify locomotor hyperactivity as a core outcome of cortical glutamatergic neuron-derived hyperactive CRD-NRG1 signaling. ErbB4 hyperactivation-directed treatment studies, such as those performed in *T-Nrg1* mice,^15^ will allow to test the predictive validity of *NC-Nrg1* mice and provide an entry point for the development of more potent treatment strategies that target specific endophenotypes in schizophrenia.

## Supplementary Material

sbab027_suppl_Supplementalry_Material

## Notes

Author contributions: T.G., M.C.S.B., and H.M. carried out biochemical and histological experiments and analyzed data. V.V. generated *Stop-Nrg1* transgenic mice. T.G. and M.M.B. designed, carried out, and analyzed behavioral experiments. C.D. performed µCT analysis. E.S., M.P, H.H., and M.Z. performed electrophysiological studies. F.A., M.J.R., K.A.N, and W.Z. contributed conceptual ideas and supervised experiments. M.H.S. designed the study, supervised experiments, and wrote the manuscript. All authors read and approved the final manuscript.
