# Supplementary material for: Hyperactivity is a Core Endophenotype of Elevated Neuregulin-1 Signaling in Embryonic Glutamatergic Networks
Source: Schizophr Bull. 2021 Apr 19;47(5):1409–20. doi: 10.1093/schbul/sbab027 (PMC8379540; doi:10.1093/schbul/sbab027)
Supplement: sbab027_suppl_Supplementalry_Material [file sbab027_suppl_supplementalry_material.docx]

**Supplemental information:**

**Suppl. Materials and Methods**

**Immunostaining on paraplast embedded sections**

Following primary antibodies were used in overnight incubations: αGFAP, rabbit, 1:500 (Dako); αHA, mouse, 1:250 (Covance); αHA, rabbit, 1:500 (Abcam); αIBA1, rabbit, 1:700 (Wako); αNRG-1a/b1/2, SC-348, rabbit, 1:100 (Santa Cruz). After addition of biotinylated secondary antibody and HRP-coupled Steptavidin (Dako LSAB2), immunoreactivity was visualized in DAB substrate solution. Sections were counterstained with Hematoxylin or dehydrated in an ascending alcohol series and mounted in Eukitt^®^ (O. Kindler).

**Immunostaining on cryo- and vibratome sections**

Primary antibodies were diluted in 1% horse serum, 0.1% Triton X-100 in 1x PBS and incubated overnight at 4°C. Following primary antibodies were used: αChaT, mouse, 1:100; αNeuN, mouse, 1:100; αNRGN, rabbit, 1:100; αGAD67, mouse, 1:100 (Chemicon); αGFAP, rabbit, 1:500 (Dako); αGFP, goat, 1:500 (Rockland); αCre, rabbit, 1:1000; αHA mouse, 1:250 (Covance); αIBA1, rabbit, 1:700 (Wako); NRG1α/β1/2 rabbit, 1:100 (Santa Cruz); αOlig2, rabbit, 1:200 (John Alberta, Harvard). After three washing steps in 1x PBS at RT secondary antibodies (anti-goat-Alexa488, 1:500; anti-mouse Alexa555, 1:500; anti-mouse Alexa649, 1:100; anti-mouse Cy2, 1:100; anti-mouse Cy3, 1:1000; anti-mouse Dylight488, 1:100; anti-rabbit Alexa488, 1:2000; anti-rabbit Alexa555, 1:500; anti-rabbit Cy2, 1:100; anti-rabbit Cy3, 1:1000; anti-rabbit Cy5, 1:100; anti-rabbit Dylight488, 1:100; Dianova) and DAPI (300 nM), diluted in 1.5% horse serum in 1x PBS, were incubated for 2h at RT. Slides were washed 3x in 1x PBS, mounted in Aqua-Poly/Mount (Polysciences, Inc.), and stored at 4°C.

Quantification of cell numbers and GFAP^+^ area in chromogenic immunostainings

Quantification was performed on coronal paraffin sections (5 µm, bregma -1.7) after immunostaining and imaging tiled overviews at 10x magnification using a Zeiss Imager.Z1 microscope. Cells were counted using the cell counter plugin for FIJI software (http://fiji.sc/Cell_Counter). GFAP^+^ area in the hippocampal grey matter was quantified using a plug-in for FIJI software (<http://www1.em.mpg.de/gfap>). Data were analyzed using Microsoft Excel and GraphPad Prism. Statistical significance was tested using a one-way ANOVA with Bonferroni’s multiple comparison test.

**Quantification of GFAP/H&E staining in post** **CT brains**

Post CT brains were infiltrated (EtOH 50% for 1h, EtOH 70% 2h, EtOH 70% 2h, EtOH 96% 1h, EtOH 96% 1h, EtOH 100% 1h, EtOH 100% 1h, isopropanol 1h, xylol 2h, xylol 2h, paraplast 2h, paraplast 2h. Paraplast embedding station Microm HMP110). Brains were embedded in paraplast blocks (Microm AP280) and stored at RT. Coronal sections (5 μm) from the anterior hippocampus (bregma -1.7) were cut on a sliding microtome (Microm HM 400), immunostained for GFAP or stained with H&E. Overview brightfield images were captured using a Zeiss Axiophot microscope with a 10x objective. Prior to image acquisition an auto-white balance was performed and a background brightfield reference image was used for online shading correction. Images were analyzed using ImageJ by cropping two vertical columns (width 500.51 μm) in the somatosensory and motor cortex. For H&E stainings, images were converted to 8-bit greyscale and thresholded to remove background signal. For GFAP stainings with H&E counterstaining, the RGB image was split into three color channels, the DAB channel was extracted, converted to 8-bit greyscale and thresholded to remove background signal. Regions of interest covering cortical layers I-IV and V/VI were quantified. RawIntDen values (sum of pixels in the ROI) were normalized to area measurements for comparability. Quantification was performed on 1-4 sections per cortical area and mouse. Data were analyzed using one-way ANOVA (GraphPad Prism 5.0).

**Protein analysis**

After western blot transfer membranes were incubated with following primary antibodies: αactin, mouse, 1:1000 (Millipore); αErbB4, rabbit, 1:1000 (Abcam); αpHER4/ErbB4 (Tyr1284), rabbit, 1:1000 (Cell Signaling); αHA, mouse, 1:250 (Covance), αNRG-1a/b1/2, rabbit, 1:500 (Santa Cruz) at 4°C ON. Membranes were washed 3x in 1x TBS-Tween, incubated in secondary antibodies (goat αmouse-HRP, goat αrabbit-HRP, 1:5000, Dianova) for 1 h at RT, washed 3x in 1x TBS-Tween, and developed with Western Lightning Plus-ECL Oxidizing reagent plus and Enhanced luminol reagent plus solutions (Perkin Elmer Life Science, Inc.).

## Electrophysiology

For slice preparation, mice (10-16 weeks old) were deeply anesthetized with isofluran before decapitation. The brain was quickly removed and immersed for 2-3 min in ice-cold cutting solution (3 mM KCl, 1.24 mM NaH_2_PO_4_, 1.3 mM MgSO_4_, 26 mM NaHCO_3_, 2.4 mM CaCl_2_, 10 mM Glucose, 220 mM Sucrose). Acute coronal PFC and hippocampal slices (300 μm) were cut with a vibratome and transferred to incubation chambers with ACSF at 32-34°C (125 mM NaCl, 2.5 mM KCl, 1.25 mM NaH_2_PO_4_, 2 mM MgSO_4_, 26 mM NaHCO_3_, 1.5 mM CaCl_2_, 10 mM Glucose), aerated with 95% O_2_ and 5% CO_2_ (3-4 ml/min).

For whole-cell patch recordings, spontaneous inhibitory postsynaptic currents (sIPSCs) were recorded from PFC neurons of layer II/III at a holding potential of −70 mV. 6‐Cyano‐7‐nitroquinoxaline‐2,3‐dione (CNQX; 10 μM), dl‐2‐Amino‐5‐phosphonopentanoic acid (DL‐AP5; 50 μM) were present to block glutamate receptor‐mediated synaptic currents. Spontaneous excitatory PSCs were recorded at a holding potential of -70 mV in the presence of 5 μM strychnine and 5 μM bicuculline. For mEPSCs or mIPSCs recordings, 0.5 μM TTX was added to the bath solution. Recording pipettes were filled with solution containing 140 mM KCl, 1 mM CaCl_2_, 10 mM EGTA, 2 mM MgCl_2_, 4 mM Na_3_ATP, 0.5 mM Na_3_GTP, 10 mM HEPES, pH 7.3. Signals with amplitudes of at least two times above the background noise were selected. Patches with a serial resistance of >10 MΩ, a membrane resistance of <0.2 GΩ, or leak currents of >200 pA were excluded. Data acquisition and analysis were done using commercially available software: pClamp 10.0 (Molecular Devices, Sunnyvale, CA), MiniAnalysis (SynaptoSoft, Decatur, GA) and Prism 6 (GraphPad, San Diego, CA). For analysis of theta rhythms, autocorrelations were performed in Clampfit 10. We calculated a value of ‘relative theta power' for each cell by summing the spectral power between 4 and 14 Hz, and dividing this by the total spectral power between 1 and 50 Hz during 10 s of sIPSC activity^1^.

Field recording electrodes were pulled from thin-walled borosilicate glass capillaries and filled with ACSF. Extracellular field potential recordings were performed using a custom-built DC amplifier. Data were digitized by a DigiData 1440A (Molecular Devices, Sunnyvale, CA, USA). Initial data analysis was done in Clampfit 10.0 Molecular Devices, Sunnyvale, CA, USA). The stimulation electrode was placed in stratum radiatum at the CA3/CA1 junction for the activation of Schaffer collaterals. The recording electrode was placed in the stratum radiatum of the CA1 region. The magnitude of fEPSPs was measured as amplitude (baseline to peak) and slope (20-80% level of the falling phase). Baseline fEPSCs were set to about 50% of maximum responses. LTP was induced by three trains separated by 20 s, each train consisting of 100 Hz stimulation for 1s. Post-train responses were measured every 20 s for 60 min. fEPSPs were filtered by a four-pole Bessel filter at a corner frequency of 2 kHz, and digitized at a sampling rate of 20 kHz using the DigiData 1400A interface Molecular Devices, Sunnyvale, CA).

Software programs Mini Analysis 6.0.3 (Synaptosoft, Decatur, GA), ClampFit 10.3 (Axon Intruments/molecular Devices), Prism 5 (GraphPad software, San Diego, CA, USA), and IBM SPSS Statistics were used for data evaluation and further statistical processing. The detection threshold of spontaneous synaptic events was set at twice the baseline noise. To exclude false events, each measurement was visually inspected and analyzed. Data are presented as mean ± standard error of the mean (SEM). Shapiro–Wilk tests were used to determine data for normally and non-normally distributed data in the individual cell analysis. Parametric unpaired Student’s *t*-test or nonparametric Kolmogorov–Smirnov test were used to determine differences between different mouse lines for normally- and non-normally distributed data, respectively. The level of statistical significance was set α = 0.05. Statistical significance is indicated as an * for *p* < 0.05.

**STED microscopy and dendritic spine analysis**

Mice were anesthetized with avertin (2,2,2-​tribromethanol; 20 µl/10 g body weight) and perfused with 4% PFA in 0.1 M PBS, pH 7.4. Brains were dissected and postfixed for 24 h in 4% PFA and 24 h in 1% PFA at 4°C. Vibratome sections were cut (50-70 µm) and mounted in Moviol. STED microscopy was performed on a custom-built STED microscope ^2^ with the following settings. EYFP was excited at 480 nm with 5.5 µW and depleted at 595 nm with 31.5 µW average power in the back-aperture of the objective (glycerol immersion, 1.3 numerical aperture). Image stacks were recorded with a pixel dwell-time of 10 µs. STED microscopy images were analyzed with SpineMagick! (Nencki Institute of Experimental Biology, Warsaw). Individual spines were marked from base to head. Initially generated spine outlines based on pixel gray value and intensity were further customized to fit spine morphology. Spine length, head width, and other parameters were saved as output for graphical analysis.

**Behavior**

Mice were group housed with a 12 h light-dark cycle (lights on at 9:00 a.m.) with ad libitum access to food and waterand transferred to the behavioral unit one week before experiments. Cage maintenance was done before and after the experimental phase. Mice were weighted before and after the experimental phase, without significant differences in body weight identified. Experiments were performed during the light phase as described before^3–6^ (see details below). Experimenter was blind to genotype throughout study. Unless stated otherwise hardware from TSE Systems (Bad Homburg) was used. Test equipment was cleaned with 70 % ethanol prior to the experiment and between trials.

Basic physical exam

Male (n= 22) and female mice (n=18) were examined at PND 73-78.

General health

Mice were individually inspected for general health (weight, fur, whiskers, genitals, etc.) and scored according to: inconspicuous (score 1), moderate changes (2), heavily burdened (3).

Reflexes

Mice were tested for eyelid, whisker and ear reflexes. Mice were held in one hand and a cotton swap was brought close to the respective feature until a reflex was elicited (twitch or closing of the eyes). The reaction was evaluated as: no reflex (score 0), difficult to elicit (1), normal reflex (2) and hyperactive reflex (3).

Grip strength

For measurement of forelimb grip strength mice were positioned horizontally from a grip bar (Alluris FMI210B2) and pulled back slowly and steadily until they released their grip. This was repeated seven times, and the peak force for the forelimb paws was measured.

Behavioral testing was performed with male mice (*wt*, n=15; *Stop-Nrg1*, n=7; *NEX-Cre*, n=22; *NC-Nrg1*, n=19) at age PND 65 to PND 110 at the beginning of behavioral analysis. Testing was performed according to the following schedule (all mice were examined in the complete test battery):

day 1 Elevated plus (not shown)

day 2 Light Dark Preference Test

day 3 Open Field

day 4 Hole Board

day 5/6 Y-Maze

day 8/9 Tail Suspension Test

day 10/11 PPI

day 23-25 Fear Conditioning

day 26 Hotplate

**Light-Dark preference test**

An in-house custom-built experimental chamber consisted of two parts, a light compartment (LC) surrounded by transparent walls and a dark compartment (DC) with dark walls. Both compartments were connected by a small open door. The test started by placing a mouse into the LC, facing towards the outside wall. The time until the mouse first entered the DC was measured (‚latency to enter DC’). From this point on, the time spend in either one of the compartments and the number of crossings was measured over a period of 5 min.

**Open field test**

Mice were allowed to explore an open field arena (size: 45 x 45 x 55 cm), with transparent walls and a grey PVC floor, for 10 min with automatic tracking by an x-y-z infrared observation system. Data were recorded using ActiMot software from TSE Systems. The following parameters were recorded: distance travelled (m), time spend in center (70 % of the area) or periphery (30 % of the area) of the arena, travel speed, time active, corner visits, and rearings.

**Hole board test**

The test was performed in the same apparatus as the open field test, only a second floor with 16 holes (diameter: 2 cm) was added to the arena. Here, x-y-z detectors monitored nose pokes. Mice were allowed to explore the arena for 10 min. The following parameters were recorded: distance travelled, number of nose pokes, and exploration time.

**Y-maze spontaneous alternation test**

The assessment of working memory was performed using a Y-shaped maze (at an angle of 120°). Mice were placed into the starting arm and allowed to explore for 10 min. The experiment was recorded with an overhead video camera. The number of arm entries (as a measure of activity) and their order was scored to calculate the percentage of alternation. Arm entry was scored when all four limbs were located within the arm.

**Tail suspension test**

Mice were hung by the tail for 6 min and the time during which they actively attempt to escape versus passivity was quantified. Mice, which manage to climb up their tail were excluded from analysis.

**Prepulse inhibition test**

Startle response was measured using the San Diego Instruments’ SR-Lab startle response system. Mice were placed in a tubular enclosure on a detector platform in the test chamber. The startle reaction to an acoustic stimulus (muscle contractions or jumping, evoking a movement of the platform and a transient force) was recorded with a computer during a time window of 100 ms (beginning with the onset of the acoustic stimulus). Mice were habituated with similar enclosures for at least 5 days prior to the experiment. With two experimental setups available, two mice were recorded at a time. Genotypes altered between recorded pairs and setups. The test started with an initial habituation phase (2 min) to 65 dB background white noise (presented throughout session), followed by baseline recording for 1 min. Startle reflexes were evoked by acoustic stimuli delivered from a loudspeaker. After baseline recordings, six pulse-alone trials using startle stimuli of 120 dB intensity and 40 ms duration were applied to decrease influence of within-session habituation and scale down the initial startle response to a stable plateau. For PPI testing, the 120 dB startle pulse of 40 ms duration was applied either alone or was preceded by a non-startling prepulse stimulus of 70, 75 or 80 dB intensity and 20 ms duration. An interval of 100 ms with background white noise was employed between each prepulse and pulse stimulus. The trials were presented in a pseudorandom order with inter-trial intervals ranging from 8 to 22 s. The amplitude of the startle response (expressed in arbitrary units, AUs) was defined as the difference between the maximum force detected during a recording window and the force measured immediately before the stimulus onset. Maximum amplitudes were averaged for each individual animal, separately for all types of trials (i.e. stimulus alone or stimulus preceded by a prepulse). PPI (expressed in %) was calculated as the percentage of the startle response using the following formula:

PPI (%) = 100 – (startle amplitude after prepulse and pulse)

(startle amplitude after pulse only) × 100

**Fear conditioning**

The Ugo Basile Series 46000 System was used for fear Conditioning. The experiment comprised three experimental trials, conducted over the course of three consecutive days. On day 1 mice were conditioned in a conditioning chamber. The setup included a square conditioning box with a 15 x 15 cm grid floor and a loudspeaker. The grid was connected to a shock-scrambler unit applying an electrical shock of defined intensity and duration. Chamber walls were covered with black and white striped panels. The chamber was placed in a soundproof box with an overhead video camera connected to a monitor. Two mice were tested at a time, in two separate setups. A white noise tone was played during the experiment. Training for the contextual and cued fear memory took place at the same time. A mouse was placed into the conditioning box and its behavior (freezing rate) was observed as an initial baseline phase of 2 min. Freezing events and durations were scored automatically by the software, the entire session was recorded by video camera for later rescoring. After the 2 min pre-exposure time (= context baseline) a tone was played for 30 sec (conditioned stimulus (CS); 10 kHz, 75 dB). Directly after the tone a mild electrical foot shock was applied (0.4 mA, 2 s duration). After a 30 sec pause the tone and shock were repeated with same intensity and duration. After the second foot shock each mouse was allowed to stay in the conditioning box for 30 sec to avoid the association of aversive stimuli with the experimenter. Subsequently, mice were placed back into their home cages. The conditioning box was cleaned with 70 % ethanol between trials. On day 2, contextual fear memory was assessed by scoring the freezing response for 2 min in the conditioning box in the absence of tone and shock. On day 3 mice were placed in a new (‘non-context’) box, i.e. a transparent plastic cylinder (36 x 20 cm) on a grey plastic floor placed in the soundproof box. Baseline freezing behavior was scored for 2 min, followed by 2 min of the cue tone (CS) and scoring of the freezing response as a measure of cued fear memory. The ‘cue’ box was cleaned with water between trials to avoid olfactory cues. The experiment was video recorded for rescoring.

**Hot plate test**

Mice were placed on a metal plate (in a plexiglass cylinder) heated at 55 °C and measuring the latency (in sec) to initiate hindpaw licking. Mice were immediately removed from the hot plate and placed on a cool metal surface. Mice that escaped from cylinder were excluded from analysis.

**Suppl. Figures**

**
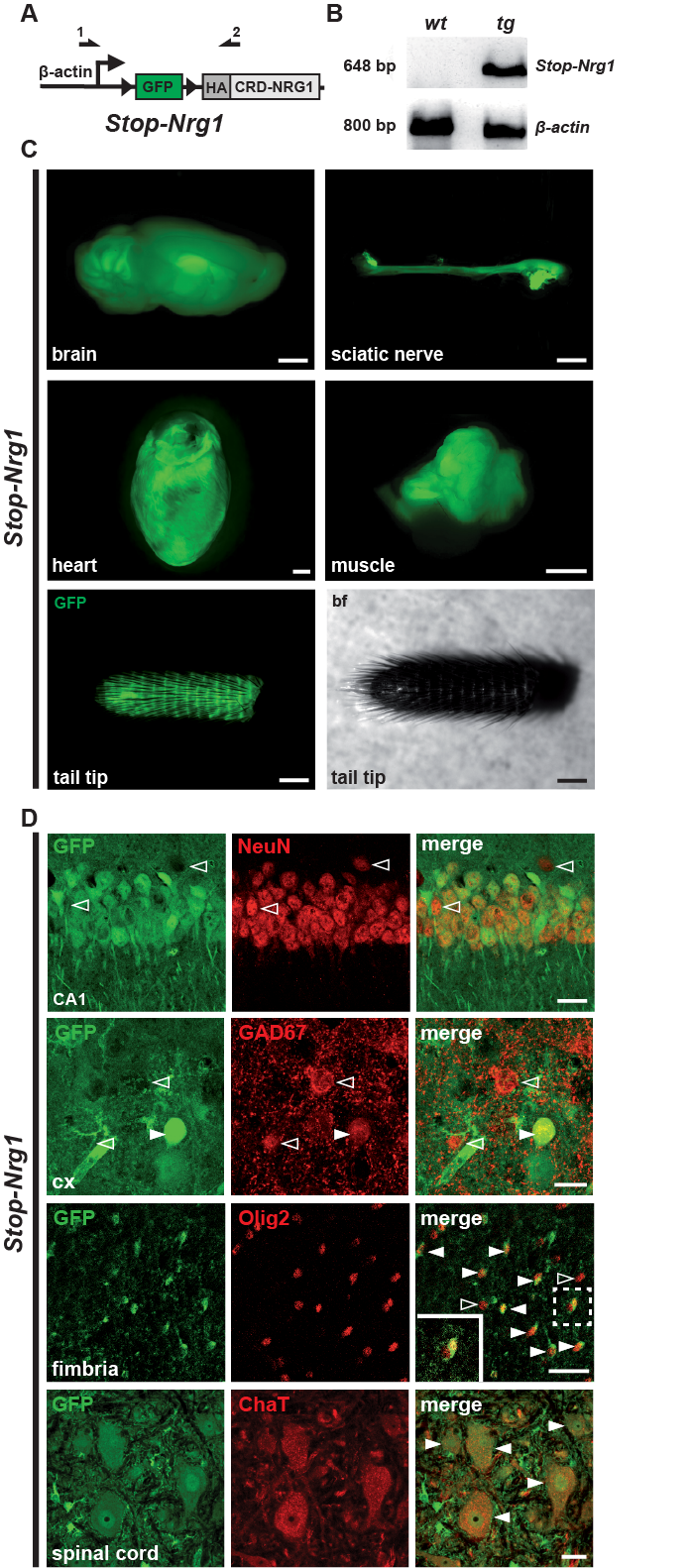
**

**Suppl. Fig. 1:** **The *Stop-Nrg1* transgene cassette is expressed in various nervous and non-nervous tissues.**

(A) Transgene cassette *Stop-Nrg1* for Cre-dependent, chicken β-actin promoter-driven N-terminally HA epitope-tagged CRD-NRG1 expression. Arrowheads mark loxP sites flanking a GFP cassette that serves as a Stop signal in the absence of Cre recombinase. Location of genotyping primers (1, 2) are indicated. (B) Genotyping PCR with primers 1 and 2 on tail genomic DNA demonstrates genomic integration of the *Stop-Nrg1* transgene. Endogenous β-actin gene amplification was used as a loading control. (C) The *Stop-Nrg1* transgene is expressed in nervous and non-nervous tissues. Images of brain, sciatic nerve, heart, muscle (quadriceps femoris), and a tail biopsy from *Stop-Nrg1* mice were produced under fluorescent light (488 nm). GFP encoded by the ‘Stop’ element can be used for fluorescent phenotyping of tail biopsies. bf, brightfield image of tail tip shown in left panel. Scale bars, 2 mm. (D) The *Stop-Nrg1* transgene is expressed in most neurons of the hippocampal CA1 region. Arrowheads mark GFP-negative neurons, most likely GABAergic interneurons. Only few GABAergic interneurons (GAD67) show GFP fluorescence, but a majority of oligodendrocytes in fimbria (Olig2; blank arrowheads mark GFP-negative oligodendrocytes) and spinal cord α-motoneurons (ChAT) express the *Stop-Nrg1* transgene. Confocal images of coronal brain sections (bregma -1.7) from *Stop-Nrg1* mice (age 3 months) following immunostaining for neural markers. Scale bars, 25 µm (Olig2, ChaT); 20 µm (NeuN); 15 µm (GAD67).

**
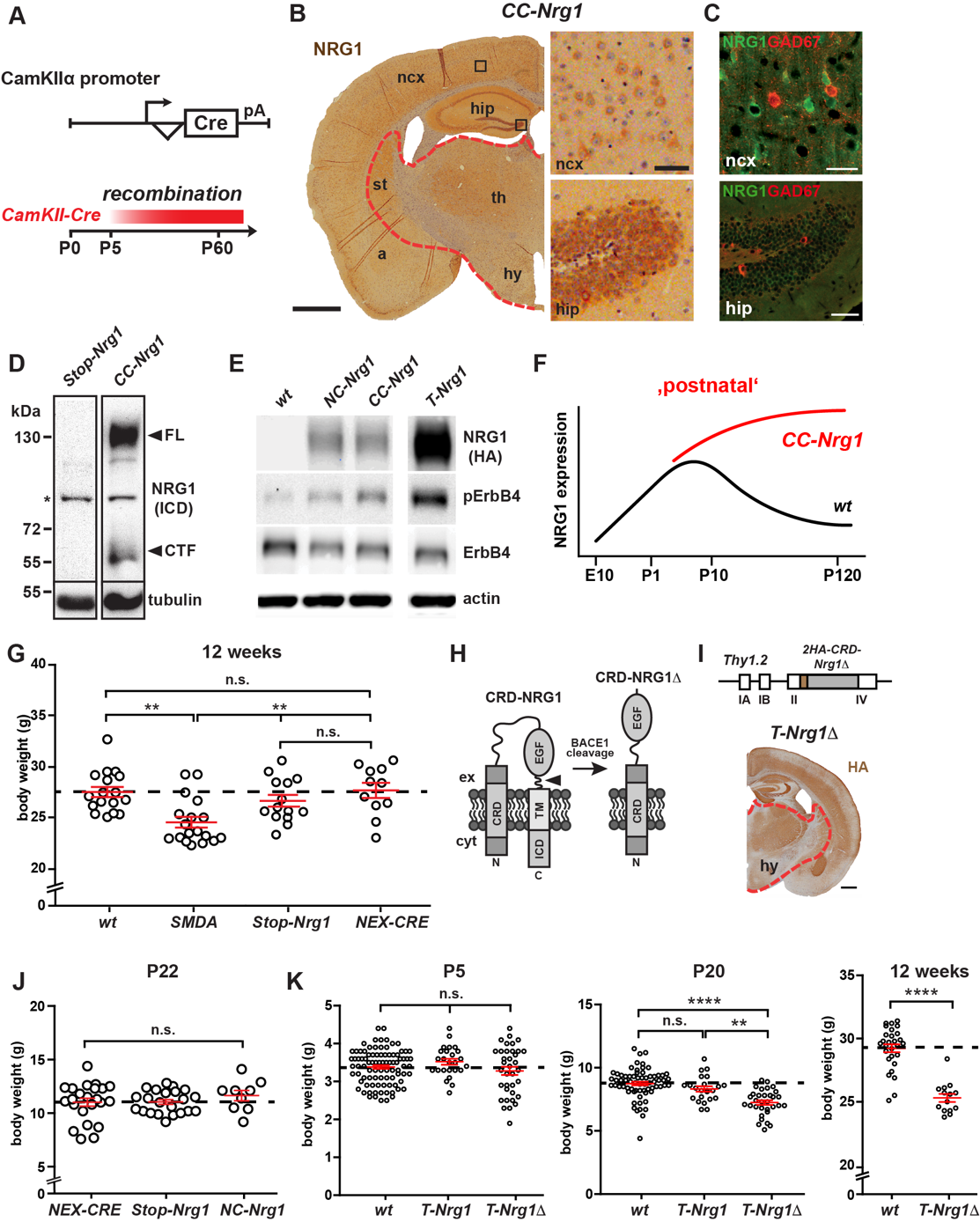
**

**Suppl. Fig. 2: Hyperstimulation of CRD-NRG1/ErbB4 signaling in glutamatergic networks and impact on body weight.**

(A) *CamKII* promotor-driven transgene directs Cre expression in *CK-Cre* driver mice starting around P5. (B) Chromogenic immunostaining with an antibody directed against the intracellular domain of NRG1 on a coronal brain section from a *CC-Nrg1* mouse (age 4 months). Zoomed images (marked by boxes) illustrate NRG1 expression in neocortical projection neurons (top) and dentate gyrus granule cells (bottom). Scale bars, 500 μm (zoom, 100 µm). a, amygdala; ncx, neoortex; hip, hippocampus; hy, hypothalamus; st, striatum; th, thalamus. (C) Fluorescent co-immunostaining for NRG1 and GABAergic marker GAD67 confirms absence of CRD-NRG1 overexpression in GABAergic interneurons of neocortex and hippocampus from *CC-Nrg1* mice.  Scale bars, 50 μm. (D) Western blot analysis of hippocampal protein lysates from *CC-Nrg1* mice and *Stop-Nrg1* controls (age 4 months). (E) Comparison of CRD-NRG1 overexpression and ErbB4 hyperphosphorylation in the hippocampus of *NC-Nrg1*, *CC-Nrg1*, and *T-Nrg1* transgenic mice. Note concomitant reduction in total ErbB4 expression. (F) Schematic (,postnatal’) expression profile illustrating CRD-NRG1 expression in *CC-Nrg1* mice (red curve) compared to wildtype (black curve). (G) Reduced body weight in mouse line SMDA (with Thy1.2 promoter-driven CRD-NRG1 expression). Normal weight in parental lines *Stop-Nrg1* and *NEX-Cre* (age 12 weeks) (one-way ANOVA with Bonferroni’s multiple comparison test; **p<0.01; n.s., not significant; n=11-18). (H) BACE1 cleavage of full-length CRD-NRG1 in the juxtamembrane stalk region (arrowhead) produces membrane bound CRD-NRG1Δ lacking the TM and ICD. (I) Construct for Thy1.2 promoter-driven expression of 2HA epitope-tagged CRD-NRG1Δ in transgenic mice. Chromogenic immunostaining for HA epitope in *T-Nrg1Δ* mouse brain. (J) Body weight is not changed in *NC-Nrg1* mice compared to parental controls at P22 (one-way ANOVA with Bonferroni’s multiple comparison test; p>0.05; n.s., not significant; n=9-23)*.* (K) Body weight is unaltered in *T-Nrg1 and T-Nrg1Δ *mice at P5 (one-way ANOVA with Bonferroni’s multiple comparison test; p>0.05; n.s., not significant; n=25-89), but reduced in *T-Nrg1Δ *mice at P20 (one-way ANOVA with Bonferroni’s multiple comparison test; ****p<0.0001; n=23-67). Profound body weight reduction in *T-Nrg1Δ *mice at 12 weeks of age (two-tailed t-test; ****p<0.0001; n=14-28).


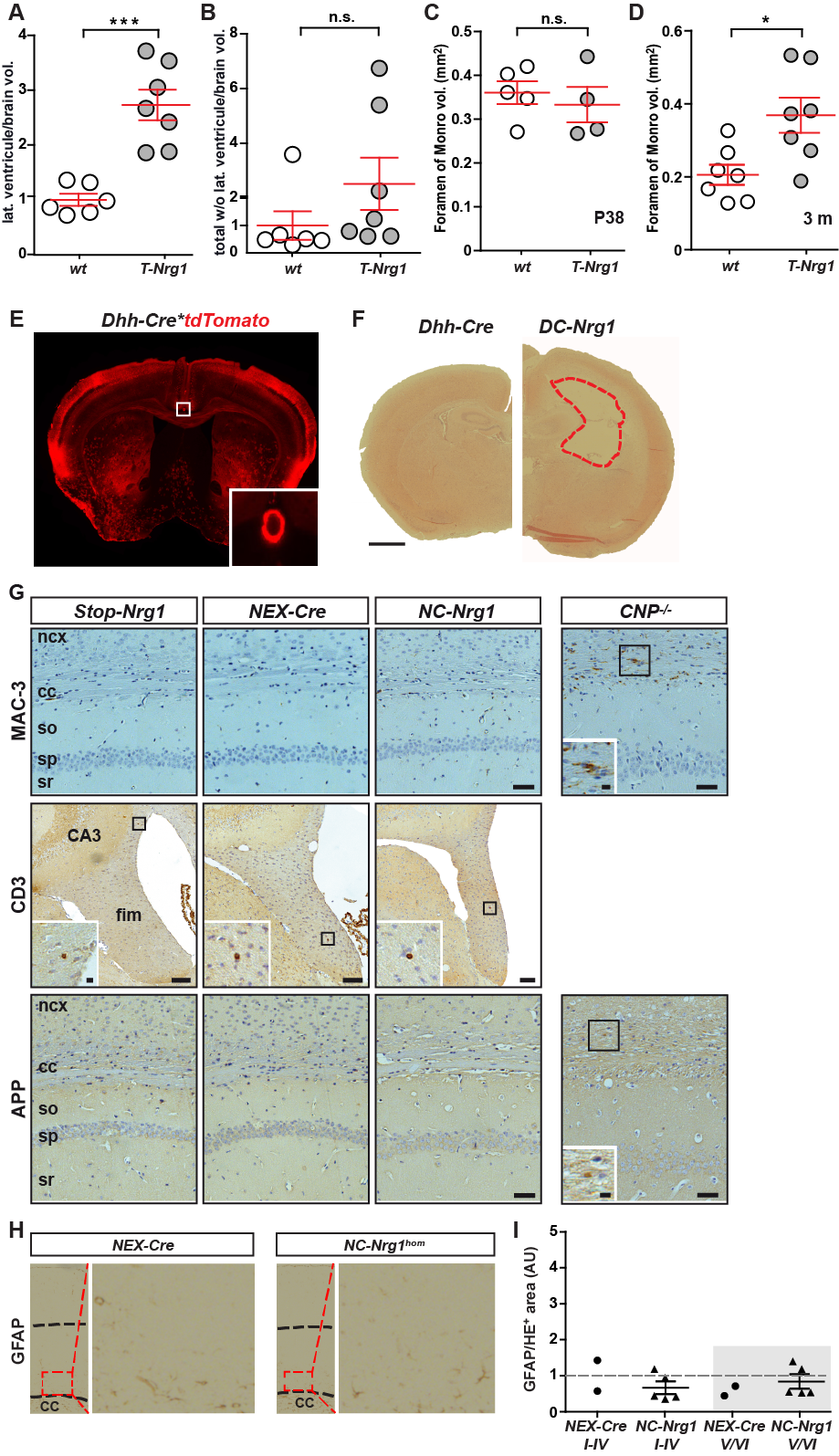


**Suppl. Fig. 3: No signs of axonal pathology and inflammation in *NC-Nrg1* mice.**

(A) Quantification of lateral ventricle volume relative to total brain volume (age 3 months). (B) Quantification of ventricle volume without lateral ventricle volume relative to total brain volume (age 3 months). (C, D) Quantification of foramen of Monroe volume at P38 (C) and 3 months of age (D). Error bars (A-E), normalized mean ± SEM. Two-tailed t-test. (*p<0.05; ***p<0.001). (E) Fluorescent micrograph of a coronal vibratome section (P30) after breeding a *Dhh-Cre* driver to a *tdTomato* reporter line ^7^. Blow up shows fluorescent blood vessel endothelial cells. (F) H&E stained post CT brain sections from a *Dhh-Cre* control and a *DC-Nrg1* mouse. Enlarged ventricle in *DC-Nrg1* brain is marked. Scalebar, 1 mm. (G)Chromogenic immunostainings on coronal brain sections (bregma -1.7) for activated microglia (MAC-3), T-cell infiltration (CD3), and axonal swellings (APP) reveal no signs of neuropathology or -inflammation in *NC-Nrg1* mice compared to parental controls (age 4 months). Insets in CD3 stainings (boxed areas in overviews) show examples of occasional CD3^+^ T-cells. Sections from cryolesioned CNP^-/-^ mouse mutants were used as positive controls. Scale bars, 50 μm (overviews), 100 μm (CD3), 10 μm (insets). (H) Immunostaining for GFAP on coronal brain sections from a *NEX-Cre* control and a *NC-Nrg1^hom^* mouse following CT analysis. (left panels) Upper dashed line separates cortical layers I-IV from V/VI, lower dashed line marks border to corpus callosum (cc). (right panels) Zoomed images (boxed in left panel). Scale bars, 100 µm (25 µm, zoom). (I) GFAP^+^ area (normalized to HE^+^ area) calculated as ratio lower/upper cortex. Values normalized to *NEXCre* controls. Note that normalization to HE^+^ area was performed using HE stained adjacent sections (not shown) and that data were pooled from *NC-Nrg1* and *NC-Nrg1^hom^* mice (n=5). Statistical analysis was not performed due to n=2 *NEX-Cre* controls.

**
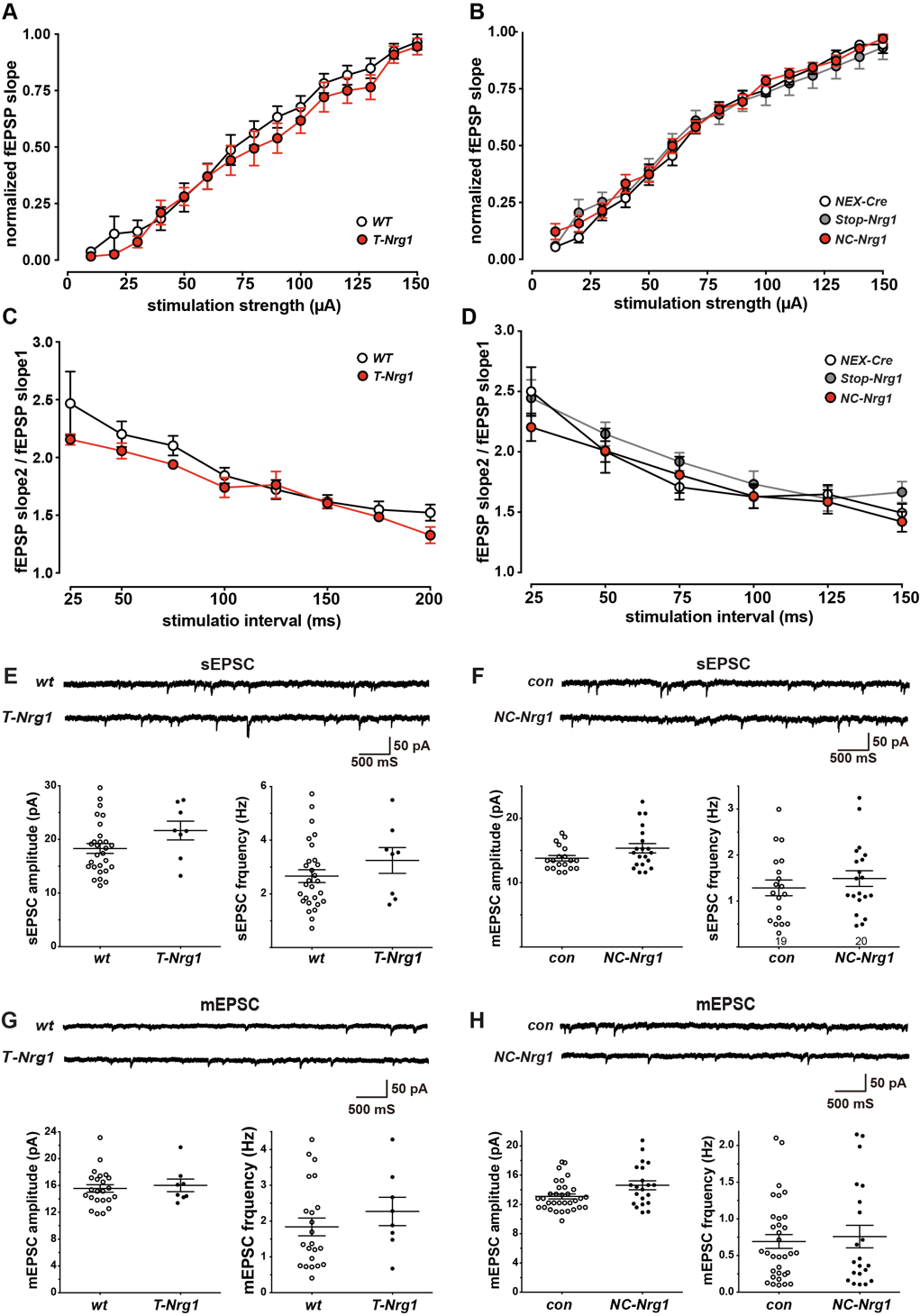
**

**Suppl. Fig. 4 Unaltered Excitatory synaptic transmission in hippocampus and prelimbic cortex of *T-Nrg1* and *NC-Nrg1* mice.**

(**A, B**) Input-output curves, as a measure of baseline excitatory synaptic transmission (depicted as fEPSP slope plotted against the stimulation strength), was not altered in the hippocampal CA1 region of *T-Nrg1* (**A**) and *NC-Nrg1* mice (**B**) compared to controls. (**C**, **D**) Paired-pulse ratios were not significantly changed in the hippocampal CA1 region of *T-Nrg1* (**C**) and *NC-Nrg1* mice (**D**) compared to controls. (**E**, **G**) top: sample traces of sEPSC (**E**) and mEPSC (**G**) recordings from pyramidal neurons in PrL of wt and *T-Nrg1* mice. Below: Both averaged amplitudes and frequencies of sEPSC (**E**) and mEPSC (**G**) recordings were not significantly altered in PrL of *T-Nrg1* mice compared to wt. (**F, H**) Top: sample traces of sEPSC (**F**) and mEPSC (**H**) recordings from pyramidal neurons in PrL of control and *NC-Nrg1* mice. Below: Both averaged amplitudes and frequencies of sEPSC (**F**) and mEPSC (**H**) recordings were not significantly altered in PrL of *NC-Nrg1* mice compared controls.

**
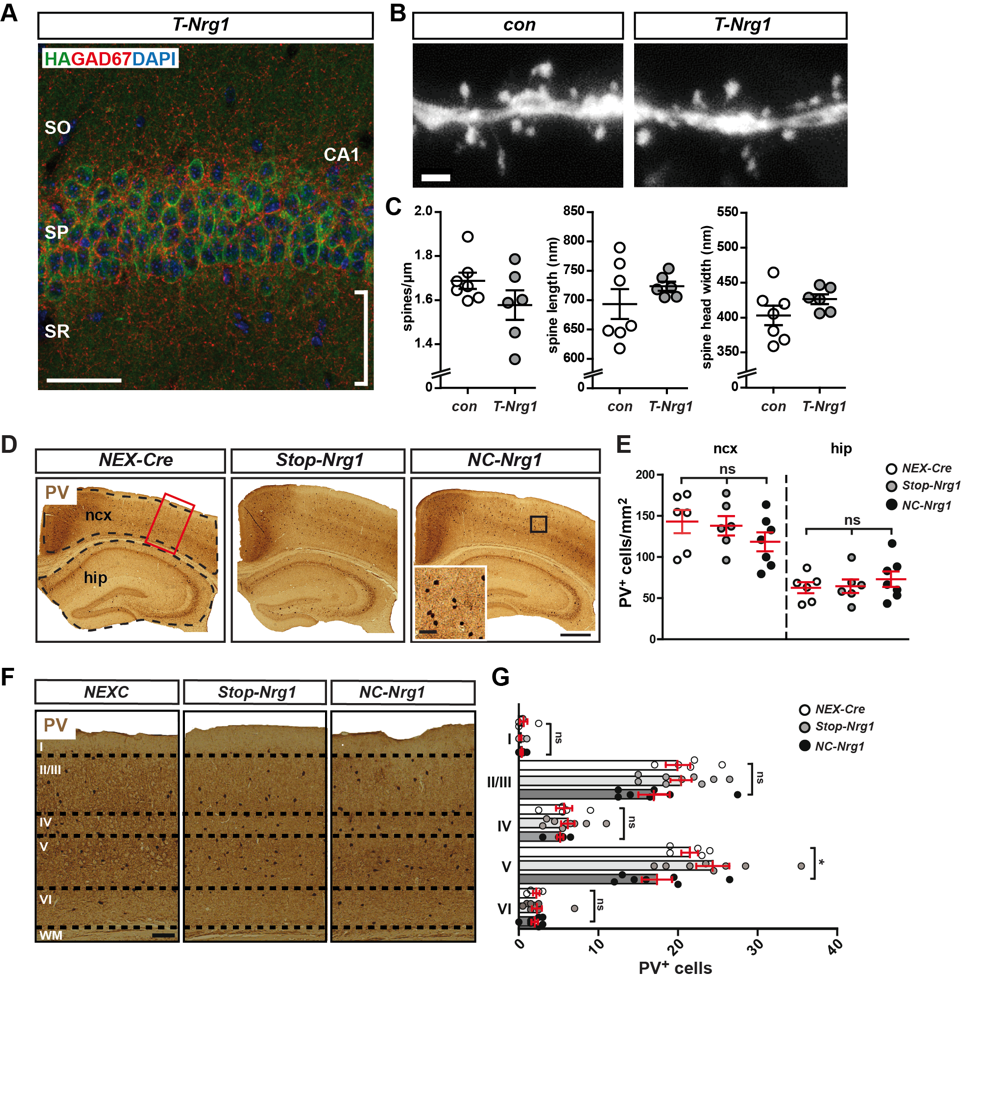
**

**Suppl. Fig. 5 Dendritic spines and PV^+^ interneurons in *T-Nrg1* and *NC-Nrg1* mice**

(A) Fluorescent immunostaining of the hippocampal CA1 region on a coronal brain section from a *T-Nrg1* mouse (age 4 months). Bracket marks analysed area in stratum radiatum. SO, stratum oriens; SP, stratum pyramidale; SR, stratum radiatum. Scale bar, 50 μm. (B) STED microscopy of hippocampal slices (stratum radiatum) from *Thy1.2-EYFP* (control) and *Thy1.2-EYFP/T-Nrg1* double transgenic mice (*T-Nrg1*; age 10 weeks). Scale bar, 1 μm. (C) Quantification of spine number, length, and head width using SpineMagick! software (control, n=7 mice; *T-Nrg1*, n=6; 4 images/mouse; 1-3 dendrites/image). n.s., not significant; multiple t-tests. (D) Chromogenic immunostainings for PV on coronal brain sections (bregma -1.7) from *NC-Nrg1*and control mice (*Stop-Nrg1*; *NEX-Cre*) at 4 months of age. Dashed lines indicate quantified areas in hippocampus and neocortex. Red box marks quantified neocortical area in F. Black box marks position of high magnification image. Scale bars, 500 μm (overview), 100 μm (inset). (E) Quantification of PV^+^ interneurons in neocortex and hippocampus. n=6-9 mice/genotype; one-way ANOVA; Bonferroni’s multiple comparison test; n.s., not significant; hip, hippocampus; ncx, neocortex. (F) Chromogenic immunostainings for PV on coronal brain sections (bregma -1.7) at 4 months of age. Cortical layers are marked by dashed lines. Scale bar, 100 μm. I–VI, cortical layers; WM, white matter. (G) Quantification of PV^+^ interneurons in somatosensory cortex columns (width 600.82 μm) from *NC-Nrg1* miceand controls (*Stop-Nrg1, NEX-Cre*). n=5-9 mice/genotype; one-way ANOVA, *p< 0.05; n.s, not significant.

**
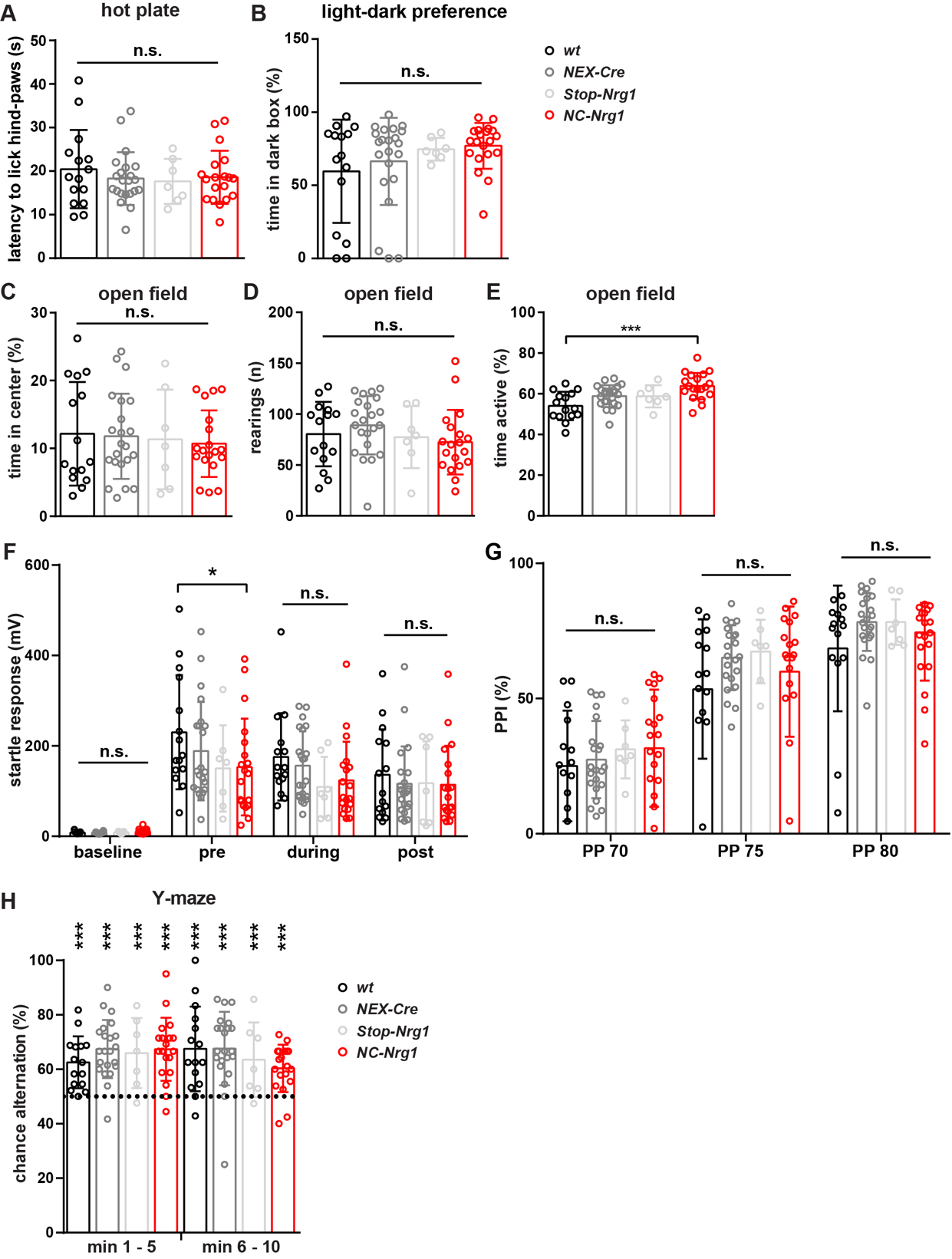
**

**Suppl. Fig. 6: Cortical-restricted CRD-NRG1 hyperstimulation in glutamatergic neurons is not associated with anxiety-like behavior or deficits in sensorimotor gating and working memory.**

(A) *NC-Nrg1* mice displayed normal pain sensitivity in the hotplate test. (B) No signs of anxiety-like behavior in light-dark preference test as time spent in dark compartment was not altered in *NC-Nrg1* mice. (C-E) *NC-Nrg1* mice showed no signs of anxiety in the open field test, i.e. unaltered time spend in the center of the arena (C) and number of rearings (D), but total time active was increased (E). (F) *NC-Nrg1* mice showed a reduced startle response prior to, but not during or after conditioning. (G) No PPI impairment in *NC-Nrg1* at prepulses of 70, 75, and 80 dB. (H) No deficits in the first and second half of the Y-maze spontaneous alternation test in *NC-Nrg1* mice. Data were analysed using ordinary one-way ANOVA with Sidak’s multiple comparisons post test (A, B, C, D, E), two-way ANOVA with Dunnett’s post test for repeated measurements (F, G) or single sample t-test against chance level alternation (H). Individual data points are shown with means ± SD. n.s., not significant, ***p<0.001, **p<0.01, *p<0.05.

|  | | ***WT*** | ***Stop-Nrg1*** | | ***Nex-Cre*** | | ***NC-Nrg1*** | |
| --- | --- | --- | --- | --- | --- | --- | --- | --- |
|  | | Mean±SD | Mean±SD | P-val. | Mean± SD | P-val. | Mean± SD | P-Val. |
| Age [days] | | 77.9±6.9 | 77.6±7.9 | - | 72.8±8.5 | - | 75.9±8.1 | - |
| Body Weight | Male | 30.4±3.9 | 28.2±5.7 | 0.46 | 28.5±1.2 | 0.28 | 29.9±3.0 | 0,85 |
|  | Female | 22.4±2.33 | 23.6±1.2 | 0.37 | 22.9±1.6 | 0.68 | 22.4±0.4 | 0,97 |
| General Body Score | | 1.0±0.0 | 1.0±0.0 | 1 | 1.3±0.5 | 0.07 | 1.0±0.0 | 1 |
| Eyelid reflex | | 1.8±0.6 | 2.0±0.0 | 0.4 | 2.0±0.0 | 0.33 | 2.0±0.0 | 0.46 |
| Ear Reflex | | 2.0±0.0 | 1.6±0.7 | 0.05 | 1.8±0.4 | 0.15 | 2.0±0.0 | 1 |
| Whiskers Reflex | | 1.9±0.3 | 1.9±0.4 | 0.88 | 2.0±0.0 | 0.28 | 2.0±0.0 | 0.42 |
| GripStrength Forelimbs [N] | | 1.2±0.1 | 1.3±0.1 | 0.11 | 1.2±0.1 | 0.57 | 1.2±0.1 | 0.89 |

**Table 1: Physical exam**

When compared to age matched *WT,* *Stop-Nrg1* and *NC-Nrg1* mice at 8-11 weeks of age showed no significant changes in body weight, general health, reflexes, and grip strength. p-values to respective wt; Graph Pad; Multiple T-Test.

**References**

1. Hu W, Zhang M, Czéh B, Flügge G, Zhang W. Stress impairs GABAergic network function in the hippocampus by activating nongenomic glucocorticoid receptors and affecting the integrity of the parvalbumin-expressing neuronal network. *Neuropsychopharmacology*. 2010;35(8):1693-1707. doi:10.1038/npp.2010.31

2. Willig KI, Steffens H, Gregor C, Herholt A, Rossner MJ, Hell SW. Nanoscopy of filamentous actin in cortical dendrites of a living mouse. *Biophys J*. 2014;106(1):L01-L03. doi:10.1016/j.bpj.2013.11.1119

3. Agarwal A, Zhang M, Trembak-Duff I, et al. Dysregulated expression of neuregulin-1 by cortical pyramidal neurons disrupts synaptic plasticity. *Cell Rep*. 2014;8(4). doi:10.1016/j.celrep.2014.07.026

4. Wehr MC, Hinrichs W, Brzózka MM, et al. Spironolactone is an antagonist of NRG1‐ERBB4 signaling and schizophrenia‐relevant endophenotypes in mice. *EMBO Mol Med*. 2017:e201707691. doi:10.15252/emmm.201707691

5. Brzózka MM, Unterbarnscheidt T, Schwab MH, Rossner MJ. OSO paradigm - A rapid behavioral screening method for acute psychosocial stress reactivity in mice. *Neuroscience*. 2016;314. doi:10.1016/j.neuroscience.2015.11.043

6. Brzózka MM, Radyushkin K, Wichert SP, Ehrenreich H, Rossner MJ. Cognitive and Sensorimotor Gating Impairments in Transgenic Mice Overexpressing the Schizophrenia Susceptibility Gene Tcf4 in the Brain. *Biol Psychiatry*. 2010;68(1):33-40. doi:10.1016/j.biopsych.2010.03.015

7. Madisen L, Zwingman TA, Sunkin SM, et al. RESOURCE A robust and high-throughput Cre reporting and characterization system for the whole mouse brain. 2010;13(1). doi:10.1038/nn.2467
